# Analysing food groups and nutrient intake in adults who met and did not meet the daily recommended vegetable intake of 350 g: the 2016 National Health and Nutrition Survey in Japan

**DOI:** 10.1017/jns.2024.5

**Published:** 2024-03-05

**Authors:** Xiaoyi Yuan, Ryoko Tajima, Mai Matsumoto, Aya Fujiwara, Tomoko Aoyama, Chika Okada, Emiko Okada, Hidemi Takimoto

**Affiliations:** 1 Department of Nutritional Epidemiology and Shokuiku, National Institutes of Biomedical Innovation, Health, and Nutrition, Osaka, Japan; 2 Division of Food Safety Information, National Institute of Health Sciences, Kawasaki City, Kanagawa, Japan

**Keywords:** dietary intake, Japanese adults, national survey, vegetables

## Abstract

This study aimed to compare the differences in the intake of food groups and nutrients between Japanese adults who consumed the recommended daily vegetable intake (350 g/day) and those who did not. Dietary information was obtained from one-day dietary records collected from the 2016 National Health and Nutrition Survey, which was conducted in 46 prefectures in Japan. The participants aged ≥20 years (*n* = 21,606; 53.8% women) were classified into the < and ≥350 g/day groups. Inter-group differences for 17 food groups and 27 nutrients were assessed as percentages of consumers (food groups only) and energy-adjusted intake (units/MJ/d or % of total energy intake). Overall, 29% of participants consumed ≥350 g/day of vegetables. The ≥350 g/day group had a higher percentage of consumers and energy-adjusted intakes for all vegetable subgroups than the <350 g/day group. For other food groups, the ≥350 g/day group had higher percentages of consumers for all food groups, except for cereals, eggs, and condiments and seasonings, which showed no significant differences. However, the ≥350 g/day group had a significantly higher energy-adjusted intake for potatoes and other tubers, mushrooms, meats, and condiments and seasonings but a significantly lower value for cereals, eggs, savoury snacks and confectionaries, and beverages. The ≥350 g/day group had a significantly higher intake of almost all (25/27) nutrients, including sodium, than the <350 g/day group. Participants with vegetable intake ≥350 g/day might have a more favourable intake of food groups and nutrients; however, watching for salt intake is necessary when promoting vegetable intake.

## Introduction

Vegetables are good sources of essential nutrients and bioactive products (e.g., polyphenols) that may protect against chronic diseases.^(1,2)^ However, the average vegetable intake worldwide is far from optimal.^(3)^ A diet low in vegetables ranks fifth among the leading dietary factors associated with the risk of developing and dying from lifestyle-related diseases.^(4)^ Food-based dietary guidelines from more than 100 countries recommend intake of vegetables (and fruits).^(5,6)^ However, countries such as the United States (US),^(7)^ United Kingdom (UK),^(8)^ and Australia^(9)^ have reported failing to meet their respective recommendations for vegetable (and fruit) intake.

In Japan, the recommended vegetable intake (350 g/day)^(10)^ was established based on the 1998 FAO/WHO guidelines, which suggested that the amount of food intake in food-based dietary guidelines should be determined based on the intake to meet the reference intake of nutrients.^(11)^ Specifically, there are four steps listed in the guidelines^(11)^: (1) identify nutrients relevant to the health problems of the target population; (2) select target foods for the nutrients identified in step (1); (3) consider the culture and socioeconomic status of the population; and (4) consider the changes that may occur in the intake of other food groups related to the changes in intake of the target food groups when the guidelines are introduced. Health Japan 21^(10)^ referred to the guidelines^(11)^ and determined that the daily vegetable intake for Japanese adults should be 350 g to meet the reference intake values for potassium, vitamin C, and dietary fibre.^(12)^ In a previous study,^(13)^ data from the 2003 National Health and Nutrition Survey in Japan (NHNSJ) were used to examine the relationship between vegetable intake and intake of other food groups and nutrients. In that study, the mean vegetable intake was 309 g/day in men and 318 g/day in women, and approximately 35% of the population consumed the recommended vegetable intake.^(13)^ However, a recent trend analysis based on NHNSJ data from 1990 to 2016 estimated that vegetable intake in the Japanese population will decrease to 238 g/day in 2040.^(14)^ The same study also projected that meeting the recommended vegetable intake would result in a 5–10% reduction in the rates of disability-adjusted life years per 100,000 population due to cancer, diabetes, and kidney diseases among the general population, and cardiovascular diseases among women aged 20–49 years.^(14)^

However, because one’s diet is composed of various foods, it is unclear how the intake of other food groups and nutrients is related to meeting the recommendations for vegetables alone. It is possible that promoting only vegetable intake, without considering its relationship with other food groups and nutrients, may be inappropriate. Studies on dietary patterns in Japanese populations have suggested that high vegetable intake is not only related to a higher intake of some healthy foods (e.g., seafood and fruits) but also sodium intake.^(15–17)^ However, no recent study has examined the intake of food groups and nutrients in relation to the recommended vegetable intake in Japan.

In this study, we aimed to compare the differences in the intake of food groups and nutrients between participants who met and those who did not meet the recommended vegetable intake (350 g/day) in Japan using the dietary data of adults aged ≥20 years obtained from the 2016 NHNSJ.

## Methods

### Data source and analytic sample

The NHNSJ is a cross-sectional household interview and examination survey that intends to represent noninstitutionalized populations aged ≥1 year across 47 prefectures in Japan. The prefectures were further grouped into 12 regions: Hokkaido, Tohoku, Kanto 1 and 2, Hokuriku, Tokai, Kinki 1 and 2, Chugoku, Shikoku, Kita-, and Minami-Kyushu.^(18)^ The present analysis used data from the most recent expanded survey, the 2016 NHNSJ.^(19)^ The sampling for the 2016 NHNSJ was based on a single-stage cluster design by randomly choosing 475 census enumeration areas based on the 2010 population census stratified by prefectures.^(19)^ Due to earthquakes or typhoons, 13 census areas in the Kumamoto and Tottori prefectures were excluded from the survey. The survey was conducted between October and November. Of 24,187 eligible households, 10,745 (response rate of 44.4%) were included in the final survey. From a total of 30,820 participants who enrolled in the 2016 NHNSJ, after excluding participants aged <20 years (*n* = 4,595), pregnant or lactating women (*n* = 256), and lack of dietary information (*n* = 4,363), 21,606 individuals aged ≥20 years were included in the present analysis.

### Dietary assessment

The dietary assessment method has been described previously^(18)^ and in the 2016 NHNSJ report.^(19)^ The dietary assessment was a one-day semi-weighed household-based dietary record. During October–November, the designated recording day was a day other than a Sunday, a public holiday, or a day with very varied intake from the usual (e.g., a wedding day). The primary household meal preparers were in charge of recording the dietary information for each household member aged ≥1 year. Trained interviewers (mainly registered dietitians) visited each sample household, distributed the booklet for the dietary record, and provided written and verbal instructions to meal preparers. For each meal, meal preparers were asked to weigh each food and beverage item used for preparation (or consumption) when possible, and they were also asked to use their household measurements (e.g., measuring cups and spoons) to record the quantity of condiments and seasonings added during cooking or at the table. When weighing the ingredients was difficult (e.g., ready-to-eat meals and provided meals), meal preparers were asked to record the portion size of the food consumed (e.g., a bowl of cooked rice) and other information, such as the restaurant’s or manufacturer’s name and to record food ingredients as detailed as possible for mixed dishes. Information on food waste and leftovers during preparation or consumption was requested. When meals were shared by household members and the food consumed by each member could not be weighed, the approximated shared portion (e.g., percentage) was recorded for each member. After the dietary record was completed, trained interviewers revisited each household (usually the following weekday) to collect and review the records, probe for missing or erroneous information, and gather further details (e.g., whole or low-fat milk, food ingredients and consumed amount of mixed dishes prepared outside the home). Trained interviewers coded food items on the dietary record based on the Standard Tables of Food Composition in Japan (STFCJ), 2010^(20)^ and assigned weights for food items recorded in portion sizes or household measures. For mixed dishes consumed outside the home with limited information on food ingredients provided, ingredients were disaggregated using published recipe books based on the dish names. Nutrient intake was calculated as consumed for each individual based on the 2010 STFCJ.^(20)^ Food recorded as cooked was converted to raw weight when a yield factor was available.^(20)^ The conversion to raw weight was applied to most vegetables, excluding fermented (or pickled) vegetables, for which the weight was used as it is. However, weight recorded as dried food (e.g., dried noodles, dried seaweeds) was converted to boiled or soaked.

The accuracy of dietary intake estimated from the household-based dietary records has been previously assessed.^(21)^ Briefly, 32 female dietetic students were trained to obtain dietary records that would serve as references. The meal preparers (mothers) from the students’ households also recorded data, which were compared with the reference data. Consequently, the relative differences in energy, protein, fat, and carbohydrate between the meal preparers and students were 6.2%, 5.7%, 6.7%, and 6.3%, respectively. The corresponding Pearson correlation coefficients were 0.90, 0.59, 0.91, and 0.90, respectively.^(21)^

### Food groups

In this study, food groups were classified based on the categories listed in the NHNSJ,^(19)^ which comprised 17 primary food groups (vegetables, potatoes and other tubers; mushrooms; seaweeds; cereals; sugar and sweeteners; legumes; nuts; fruits; seafood; meats; eggs; milk and dairy products; fats and oils; savoury snacks and confectionaries; beverages; and condiments and spices), as well as 26 secondary and 85 tertiary groups. In this study, the analyses were performed at the primary, secondary, and tertiary levels for vegetables and potatoes and other tubers, and at primary and, when possible, secondary levels for the other 15 food groups. In total, 17 primary, 21 secondary, and 16 tertiary groups were included in the analysis (Table S1).

### Diet quality

Diet quality was tentatively assessed as one’s intake of the total number of nutrients (*n* = 27) that met the Dietary Reference Intakes (DRIs) for the Japanese according to sex and age groups^(22)^ (Table S2). We acknowledged that one-day nutrient intake was not appropriate to compare to the DRIs that were used for assessing habitual intake. However, because a validated diet quality scale was unavailable in Japan during the data analysis, the comparison to the DRIs was only used to serve as a snapshot of the overall dietary intake between groups rather than assessing nutrient intake adequacy. A similar method has been used in a previous Japanese study.^(23)^ The intakes of protein, total fat, saturated fatty acids (SFA), and carbohydrates were calculated as a percentage of total energy intake for comparison with the DRIs. Other nutrient intake was adjusted to sex- and age-specific estimated energy requirement (EER) for physical activity level II (normal) as follows: EER-adjusted intake (unit/d) = EER (MJ/d) × reported intake (unit/d)/reported energy intake (MJ/d). The EER-adjusted intake was used for two reasons. First, dietary intake may differ between individuals owing to their sex and body composition; consequently, people with a higher daily intake may also easily meet the DRIs, irrespective of the dietary composition. Second, self-reported dietary assessments are prone to be misreported. Therefore, instead of comparing nutrient intake to the DRIs based on the reported energy intake, EER-adjusted values were used.^(24)^

### Covariates

Body height and weight were measured to the nearest 0.1 cm and 0.1 kg, respectively, in barefoot conditions with light clothing at the examination sites or by trained reviewers in household settings. If direct measurement was impossible, self-reported values (*n* = 5,750) were obtained.^(18,19)^ Body mass index (BMI) was calculated as weight (kg) divided by height in metres squared (m^2^). Participants with missing BMI information were not excluded from the analysis. Instead, a missing category was created to consider the presumed difference in vegetable intake. Therefore, BMI was divided into four categories for assessing weight status: <18.5, 18.5 – 24.9, and ≥25.0 kg/m^2^ and missing. Information regarding gender, age, smoking experience, and drinking habits was obtained using a self-reported questionnaire.^(18,19)^ Age was categorised into the following seven groups: 20–29, 30–39, 40–49, 50–59, 60–69, 70–79, and ≥80 years. Smoking experience (yes, no, and missing) was categorised based on the question, ‘Do you smoke cigarettes?’^(18,19)^; participants who answered, ‘I smoke every day’ and ‘I used to smoke but have not smoked for ≥1 month’ were classified as experienced smokers (i.e., yes). Those who answered ‘do not smoke’ were classified as non-experienced smokers (i.e., no). Drinking habits were assessed based on the question, ‘how many days a week do you drink alcohol (*sake*, *shochu,* Western-style alcohol, etc.)?’^(18,19)^; participants who answered ‘every day’, ‘5–6’, ‘3–4’, and ‘1–2 days a week’ were classified as having drinking habits (i.e., yes), while those who answered ‘1–3 days a month’, ‘rarely’, ‘quit’, and ‘do not [cannot] drink’ were classified as not having drinking habits (i.e., no).^(25)^ A missing category was also created for smoking experience and drinking habits, as was the case for BMI.

### Statistical analyses

Participants were classified into two groups: those who consumed <350 g/day of vegetables (excluding potatoes and other tubers, mushrooms, and seaweeds) and those who consumed ≥350 g/day of vegetables. The self-reported crude value of vegetable intake was used to classify participants because the recommended value was irrespective of sex, age, or body composition.^(10)^ To compare basic characteristics, categorical variables were expressed as numbers and percentages, and the chi-square test was used to compare inter-group differences. Continuous variables were expressed as means and standard deviations (SD), and inter-group differences were compared using an independent-sample *t*-test.

For the intake of vegetables and other food groups, multivariable logistic regression was first used to calculate the covariate-adjusted percentage of consumers (i.e., intake >0 g/day) in each food group, which was then tested using the Wald chi-square test. Covariates included sex, age, region, weight status, smoking experience, and drinking habits (covariates remained the same for each multivariate analysis). The mean intake (based on the energy density method)^(26)^ was calculated for each food group for consumers only. The distributions of intake of various food groups were skewed (observed using histograms and Q-Q plots); therefore, generalised linear models based on gamma distributions with log-link functions were used to compute the covariate-adjusted least-squares mean intake of a food group.^(27)^ Log-transformed intake was back-transformed to the original scale.

No analysis was performed on the percentage of consumers of nutrients because almost all participants (missing, *n* < 49) consumed all the nutrients included in the analysis. The distributions of nutrient intake (adjusted using the energy density method) were nearly normal; therefore, general linear models based on normal distributions with the original scale were used for the analysis. The least-squares means of all energy-adjusted intake for food groups (for the consumers only) and nutrients are shown as a mean with the 5^th^ and 95^th^ percentiles of the covariate-adjusted distributions.

For dietary quality, the total number of nutrients in each participant’s EER-adjusted intake that met the DRIs was calculated and divided into tertiles (i.e., number of nutrients T1:17–27; T2:13–16; and T3:0–12 nutrients). The number of nutrients that met the DRIs was highest in the first tertile (T1). In the <350 g/day and ≥350 g/day groups, the covariate-adjusted proportion of participants for each tertile and the proportion of participants that met the DRI for each nutrient were calculated using multivariate logistic regression and compared using the Wald chi-square test. Statistical analyses were performed using SAS version 9.4 (SAS Institute Inc., Cary, NC, USA). A two-tailed *P*-value of < 0.001 was considered statistically significant.^(28)^

### Additional analyses

Most participants from this study were below the recommended vegetable intake, and the diet quality of participants with very low vegetable intake (e.g., <70 g/d) may differ from those close to the recommended intake. Based on this, adherence to the DRI was further tested by categorising participants into eight groups using a serving size of 70 g^(29)^ from <70 g/d to ≥490 g/d. Linear trends were tested using the Cochran–Armitage test.^(30)^ Between-group differences from the reference group (280–350 g/day) were tested based on multivariable logistic regression adjusted to the covariates described in the previous section.

Given the low fibre content of vegetable juice and high sodium content of fermented (or pickled) vegetables,^(20)^ the analysis was repeated by excluding vegetable juice and fermented (or pickled) vegetables to assess whether the exclusion may reveal different results.

## Ethics statement

This study was conducted according to the guidelines laid down in the Declaration of Helsinki; all individual participants provided verbal informed consent. Verbal consent was witnessed and formally recorded. No institutional review board approval was required because only anonymized data were used, according to the Ethical Guidelines of Epidemiological Research by the Ministry of Education, Culture, Sports, Science and Technology and the Ministry of Health, Labour and Welfare. The Ministry of Health, Labour and Welfare approved the 2016 NHNSJ for the present analysis under the Statistics Act.

## Results

The mean age of the 21,606 participants included in this analysis was 58.0 (SD, 17.5) years, and the mean BMI was 23.1 (SD, 3.6) kg/m^2^. Less than one-third (28.8%) of the participants had a vegetable intake of ≥350 g/day (Table 1). About half (51.6%) of the participants were aged ≥60 years, and 46.4% were from Eastern Japan (i.e., Hokkaido, Tohoku, Kanto 1 and 2, and Hokuriku). Participants who were men aged 60–79 years and recruited from Eastern Japan (Figure S1) were more likely to have a vegetable intake of ≥350 g/day. Participants with missing values for weight status, smoking experience, and drinking habits were less likely to meet recommendations. The mean crude vegetable intake was 281 (SD, 174) g/day for all participants and 195 (SD, 88) and 495 (SD, 146) g/day in the < 350 and ≥ 350 g/day groups, respectively.

**Table 1.** Basic characteristics of all participants and by groups of meeting the Japanese recommendation of vegetable intake (350 g/day), adults ≥20 years, 2016 NHNSJ (*n* 21,606)^†^

	All (*n* 21606)	<350 g/day (*n* 15391)	≥350 g/day (*n* 6215)
Gender, *n* (%)	*		
Men	9987 (46.2)	6939 (45.1)	3048 (49.0)
Women	11619 (53.8)	8452 (54.9)	3167 (51.0)
Age group (year), *n* (%)	*		
20–29	1422 (6.6)	1121 (7.3)	301 (4.8)
30–39	2397 (11.1)	1886 (12.3)	511 (8.2)
40–49	3382 (15.7)	2667 (17.3)	715 (11.5)
50–59	3263 (15.1)	2359 (15.3)	904 (14.5)
60–69	4948 (22.9)	3229 (21.0)	1719 (27.7)
70–79	3936 (18.2)	2475 (16.1)	1461 (23.5)
≥80	2258 (10.5)	1654 (10.7)	604 (9.7)
Region, *n* (%)^‡^	*		
Hokkaido	377 (1.7)	266 (1.7)	111 (1.8)
Tohoku	2861 (13.2)	1920 (12.5)	941 (15.1)
Kanto 1	1816 (8.4)	1249 (8.1)	567 (9.1)
Kanto 2	2810 (13.0)	1933 (12.6)	877 (14.1)
Hokuriku	2156 (10.0)	1524 (9.9)	632 (10.2)
Tokai	2060 (9.5)	1592 (10.3)	468 (7.5)
Kinki 1	1151 (5.3)	853 (5.5)	298 (4.8)
Kinki 2	1282 (5.9)	944 (6.1)	338 (5.4)
Chugoku	2308 (10.7)	1699 (11.0)	609 (9.8)
Shikoku	1993 (9.2)	1389 (9.0)	604 (9.7)
Kita-Kyushu	1586 (7.3)	1140 (7.4)	446 (7.2)
Minami-Kyushu	1206 (5.6)	882 (5.7)	324 (5.2)
Weight status (kg/m^2^), *n* (%)^§^	*		
<18.5	1307 (6.0)	950 (6.2)	357 (5.7)
18.5–24.9	11105 (51.4)	7852 (51.0)	3253 (52.3)
≥25.0	4376 (20.3)	3021 (19.6)	1355 (21.8)
Missing	4818 (22.3)	3568 (23.2)	1250 (20.1)
Smoking experience, *n* (%)^||^	*		
Yes	5495 (25.4)	4131 (26.8)	1364 (21.9)
No	15722 (72.8)	10962 (71.2)	4760 (76.6)
Missing	389 (1.8)	298 (1.9)	91 (1.5)
Drinking habits, *n* (%)^¶^			
Yes	7793 (36.1)	5487 (35.7)	2306 (37.1)
No	13415 (62.1)	9601 (62.4)	3814 (61.4)
Missing	398 (1.8)	303 (2.0)	95 (1.5)
Energy intake (MJ/d), mean (SD)	7.85 (2.31)	7.47 (2.20)	8.78 (2.32)*
Vegetables intake (g/day), mean (SD)^††^	281 (174)	195 (88)	495 (146)*
Vegetables (not including vegetable juices and fermented [or pickled] vegetables) intake (g/day), mean (SD)	259 (163)	183 (87)	449 (152)*
Potatoes and other tubers, mushrooms, and seaweeds (g/day), mean (SD)	85 (81)	75 (73)	109 (94)*
Total intake of vegetable, potatoes and other tubers, mushrooms, and seaweeds (g/day), mean (SD)	366 (210)	270 (125)	604 (185)*

NHNSJ, National Health and Nutrition Survey in Japan; SD, standard deviation.

† Data are presented as *n* (%) for categorical variables and mean (SD) for continuous variables.

‡ For each region, prefectures are included as follows: Hokkaido: Hokkaido; Tohoku: Aomori, Iwate, Miyagi, Akita, Yamagata, Fukushima; Kanto 1: Saitama, Chiba, Tokyo, Kanagawa; Kanto 2: Ibaraki, Tochigi, Gunma, Yamanashi, Nagano; Hokuriku: Niigata, Toyama, Ishikawa, Fukui; Tokai: Gifu, Aichi, Mie, Shizuoka; Kinki 1: Kyoto, Osaka, Hyogo; Kinki 2: Nara, Wakayama, Shiga; Chugoku: Shimane, Okayama, Hiroshima, Yamaguchi; Shikoku: Tokushima, Kagawa, Ehime, Kochi; Kita-Kyushu: Fukuoka, Saga, Nagasaki, Oita; Minami-Kyushu: Miyazaki, Kagoshima, and Okinawa.

§ Body mass index, as weight (kg) divided by height squared (m^2^).

|| Based on the question regarding smoking status (‘Do you smoke?’). ‘Yes’ included those who answered ‘everyday’, ‘sometimes’, or ‘smoked in the past, but have not smoked for more than a month’; ‘no’ included those who answered ‘do not smoke’.

¶ Based on the question regarding the frequency of alcohol intake (i.e., ‘How often do you drink alcohol (e.g., sake, shochu, beer, wine, etc.) in a week?’). ‘Yes’ included those who answered ‘everyday’, ‘5–6 days/week’, ‘3–4 days/week’, and ‘1–2 days/week’; ‘No’ included those who answered ‘1–3 days/month’, ‘rarely’, ‘quit’, ‘do not drink’.

†† Vegetables include green-yellow and other vegetables (e.g., cabbage), vegetable juices, and fermented (or pickled) vegetables, but not potatoes, tubers, mushrooms, or seaweeds.

* *P* < 0.001. Differences between groups were analysed using the chi-square test for categorical variables and the independent-samples *t*-test for continuous variables.

With respect to vegetables, in comparison with the <350 g/day group, the ≥350 g/day group had a significantly higher percentage of consumers (except for total vegetables) and energy-adjusted intake (g/MJ/d) (Table 2). Specifically, the energy-adjusted mean intake of green-yellow vegetables, other vegetables, and fruit juices in the ≥350 g/day group was ≥two-fold larger than that in the <350 g/day group, whereas a small inter-group difference (1.2 times) was noted for fermented (or pickled) vegetables. Although the ≥350 g/day group had a significantly higher percentage of consumers of tubers, mushrooms, and seaweeds, the energy-adjusted intake was not significantly different between the subgroups regarding tubers (i.e., sweet potatoes, potatoes, and other tubers) or seaweeds (Table 2).

**Table 2.** Percentage of consumers and intake (g/MJ/d) of vegetables, potatoes and tubers, mushrooms, and seaweeds by groups of meeting the Japanese recommendation of vegetable intake (350 g/day), adults ≥20 years, 2016 NHNSJ (*n* 21,606)^†^

	Percentage of consumers		Energy-adjusted intakes					
	<350 g/day (*n* 15391)	≥350 g/day (*n* 6215)	<350 g/day (*n* 15391)			≥350 g/day (*n* 6215)		
			P5	Mean	P95	P5	Mean	P95
Food groups included in the recommendation								
Vegetables	99.4%	100.0%	22.9	27.7	32.9	49.7	59.7	70.3*
Green-yellow vegetables	90.4%	98.0%*	7.1	9.5	12.3	14.3	19.2	24.7*
Tomatoes	25.4%	46.0%*	3.9	5.8	8.3	6.0	9.1	12.8*
Carrots	62.8%	78.7%*	2.6	3.2	3.9	4.0	4.9	5.9*
Spinach	15.0%	21.2%*	4.7	6.6	9.2	6.0	8.5	11.5*
Bell peppers	19.2%	32.3%*	2.2	2.8	3.8	2.7	3.6	4.8*
Other green-yellow vegetables	65.0%	81.7%*	4.0	5.4	7.2	6.9	9.5	12.3*
Other vegetables	97.5%	99.9%*	15.0	17.6	20.2	29.9	35.1	40.3*
Cabbages	44.2%	61.7%*	5.8	7.1	8.4	8.9	10.8	12.8*
Cucumbers	27.2%	36.6%*	2.7	3.6	4.7	3.7	5.0	6.5*
Daikon radish	36.7%	55.8%*	6.2	7.7	9.6	8.9	11.2	13.9*
Onions	61.0%	73.4%*	4.9	5.8	6.7	6.9	8.2	9.6*
Chinese cabbage	20.4%	31.7%*	6.4	8.1	10.3	10.2	12.7	16.2*
Other light-coloured vegetables	80.8%	91.3%*	4.8	6.3	7.5	8.5	11.1	13.3*
Vegetable juices	5.3%	19.0%*	6.8	10.4	15.2	14.6	21.3	31.9*
Fermented (or pickled) vegetables	38.8%	44.8%*	1.8	2.9	4.7	2.2	3.6	5.5*
Fermented (or pickled) leafy vegetables	7.9%	11.3%*	2.5	3.9	5.7	3.0	4.5	6.5*
Fermented (or pickled) radish or other vegetables	34.2%	38.6%*	1.5	2.5	3.9	1.7	2.9	4.5*
Food groups not included in the recommendation								
Potatoes and other tubers	71.2%	76.0%*	7.4	9.4	12.0	8.2	10.4	13.0*
Tubers	61.7%	68.0%*	8.6	10.4	12.6	9.2	11.1	13.5*
Sweet potatoes	11.6%	14.4%*	5.0	7.4	10.4	5.0	7.3	10.3
Potatoes	35.4%	38.9%*	7.6	8.6	9.7	7.8	8.8	10.0
Other tubers	32.6%	38.1%*	6.0	7.7	9.6	6.2	8.0	9.9
Starches	23.7%	23.2%	0.7	1.0	1.5	0.8	1.2	1.7*
Mushrooms	50.1%	62.3%*	3.3	4.1	4.9	4.0	4.9	6.0*
Seaweeds	57.2%	61.0%*	2.0	2.6	3.4	2.1	2.7	3.5

NHNSJ, National Health and Nutrition Survey in Japan; P5, 5th percentile; P95, 95th percentile.

† Adjusted percentages of consumers with intake >0 g/day based on logistic regression and adjusted intakes and distributions of consumers based on general linear models (gamma distribution and log-link function). Covariates were adjusted for sex (men or women), age (20–29, 30–39, 40–49, 50–59, 60–69, 70–79, and ≥80 years), region (Hokkaido, Tohoku, Kanto 1 and 2, Hokuriku, Tokai, Kinki 1 and 2, Chugoku, Shikoku, and Kita- and Minami-Kyushu), weight status (<18.5, 18.5–24.9, ≥25.0 kg/m^2^, and missing), smoking experience (yes, no, and missing), and drinking habits (yes, no, or missing). Intake values are shown as the mean and 5th and 95th percentiles of the adjusted distribution in the original scale.

* *P* < 0.001 between groups < and ≥ 350 g/day. Wald Chi-square test obtained from logistic regression for testing the between-group difference of the percentage of consumers, and *t*-test for testing the between-group difference of the least-square means obtained from general linear models.

For other food groups, only wheat products had significantly lower values in terms of the percentage of consumers and energy-adjusted intake in the ≥350 g/day group than in the <350 g/day group (Table 3). The energy-adjusted intake of cereals (and rice products) and eggs was significantly lower in the ≥350 g/day group than in the <350 g/day group, despite no significant difference in the percentage of consumers. Although almost all participants in the two groups consumed condiments and seasonings, the energy-adjusted intake was significantly higher in the ≥350 g/day group than in the <350 g/day group (mean [5^th^–95^th^ percentile]: 13 [11–14] and 14 [12–15] g/MJ/d, respectively). For the food groups that had a significantly higher percentage of consumers in the ≥350 g/day group than in the <350 g/day group, the energy-adjusted intake was only significantly higher for meats (and red meat) in the ≥350 g/day group, and a significantly lower intake for other cereals, fruit juices, savoury snacks and confectionaries, and beverages (and alcoholic and non-alcoholic beverages). There were no significant differences in the energy-adjusted intakes of sugar, legumes, nuts, total (and whole) fruits, fats and oils, seafood (unprocessed and processed seafood), poultry, and milk and dairy products.

**Table 3.** Percentage of consumers and intake (g/MJ/d) for other food groups by groups of meeting the Japanese recommendation of vegetable intake (350 g/day), adults ≥20 years, 2016 NHNSJ (*n* 21,606)^†^

	Percentage of consumers		Energy-adjusted intakes					
	<350 g/day (*n* 15391)	≥350 g/day (*n* 6215)	<350 g/day (*n* 15391)			≥350 g/day (*n* 6215)		
			P5	Mean	P95	P5	Mean	P95
Cereals	99.7%	99.8%	50.3	57.9	67.1	43.0	49.5	57.2*
Rice products	95.9%	96.8%	36.3	44.1	53.7	31.6	38.4	46.6*
Wheat products	83.3%	80.1%*	14.4	16.8	19.0	11.7	13.5	15.2*
Other cereals	11.0%	14.3%*	7.7	12.3	18.5	6.4	10.2	15.0*
Sugar and sweeteners	75.2%	80.3%*	0.8	1.1	1.5	0.8	1.1	1.4
Legumes	73.0%	82.3%*	9.2	11.0	12.9	9.8	11.7	13.6
Soybeans	71.9%	81.4%*	9.2	10.9	12.8	9.8	11.5	13.4
Other beans	4.9%	6.0%	2.5	3.7	5.0	2.4	3.5	4.8
Nuts	27.0%	37.5%*	0.8	1.1	1.5	0.8	1.1	1.4
Fruits	59.7%	74.2%*	12.3	19.4	27.9	12.9	21.0	29.3
Whole fruits	54.1%	70.0%*	12.1	19.1	27.5	12.7	20.5	28.2
Jams	8.4%	10.6%	1.3	1.7	2.4	1.2	1.6	2.2
Fruit juices	9.7%	14.1%*	4.0	11.5	24.4	2.4	7.1	15.1*
Seafood	80.0%	86.9%*	9.0	11.4	14.1	9.0	11.5	14.1
Unprocessed seafood	51.8%	57.0%*	8.8	10.6	12.7	8.7	10.6	12.6
Processed seafood	60.8%	69.3%*	4.7	5.9	7.4	4.5	5.7	7.0
Meats	88.4%	91.8%*	8.9	12.4	16.6	9.8	12.9	17.6*
Red meat	80.0%	83.6%*	7.4	9.5	11.9	8.1	10.1	12.7*
Poultry	35.6%	36.7%*	7.0	8.9	11.3	6.9	8.6	11
Other meats^‡^	2.0%	2.6%	5.4	8.4	11.9	4.7	7.2	10.2
Eggs	76.5%	77.3%	5.4	6.4	7.4	5.0	5.9	6.8*
Milk and dairy products	68.3%	75.6%*	13.3	19.6	27.2	12.9	19.4	26.2
Fats and oils	88.2%	91.3%*	1.2	1.5	1.8	1.1	1.4	1.7
Savoury snacks and confectionaries	40.5%	45.6%*	6.2	7.5	9.0	4.8	5.8	7.0*
Beverages	94.4%	96.2%*	68.1	93.8	124.2	61.3	83.7	110.3*
Alcoholic beverages	48.6%	55.5%*	1.9	20.9	70.5	1.6	17.7	57.9*
Non-alcoholic beverages	89.3%	91.3%*	62.1	82.7	102.6	55.2	74.1	93.4*
Condiments and seasonings	99.9%	100.0%	10.9	12.5	13.9	11.8	13.5	14.9*

NHNSJ, National Health and Nutrition Survey in Japan; P5, 5th percentile; P95, 95th percentile.

† Adjusted percentages of consumers with intake >0 g/day based on logistic regression and adjusted intakes and distributions of consumers based on general linear models (gamma distribution and log-link function). Covariates were adjusted for sex (men or women), age (20–29, 30–39, 40–49, 50–59, 60–69, 70-79, and≥80 years), region (Hokkaido, Tohoku, Kanto 1 and 2, Hokuriku, Tokai, Kinki 1 and 2, Chugoku, Shikoku, and Kita- and Minami-Kyushu), weight status (<18.5, 18.5–24.9, ≥25.0 kg/m^2^, and missing), smoking experience (yes, no, and missing), and drinking habits (yes, no, or missing). Intake values are shown as the mean and 5th and 95th percentiles of the adjusted distribution in the original scale.

‡ Included offal and other meat (e.g., frogs).

* *P* < 0.001 between groups < and ≥350 g/day. Wald Chi-square test obtained from logistic regression for testing the between-group difference of the percentage of consumers, and *t*-test for testing the between-group difference of the least-square means obtained from general linear models.

The energy-adjusted intake (% energy or unit/MJ/d) was significantly higher in the ≥350 g/day group for all nutrients, including sodium, than in the <350 g/day group, except for carbohydrates (which was significantly lower in the ≥350 g/d group) and vitamin B_12_ (Table 4). Although a significant difference was noted in SFA intake, the mean values of the two groups were similar (7.1% energy). Based on the one-day dietary record, more participants in the ≥350 g/day (59%) group consumed a larger number (17 – 27) of nutrients as per the DRIs than those in the <350 g/day (24%) group (Table 5). In line with the energy-adjusted intake, the ≥350 g/day group had a higher percentage (median, 63.1%; range, 31.2 – 99.3%) of participants who met the DRIs than those in the < 350 g/day group (median, 50.6%; range, 11.7 – 97.1%) for almost all nutrients. However, significantly lower rates were observed for SFA and sodium in the ≥350 g/day group (52.3% and 6.4%, respectively) than in the <350 g/day group (52.9% and 10.3%, respectively). No inter-group differences were observed for total fat and carbohydrates. The crude intakes of food groups and nutrients are summarised in Tables S3–S5.

**Table 4.** Energy-adjusted nutrient intake by groups of meeting the Japanese recommendation of vegetable intake (350 g/day), adults ≥20 years, 2016 NHNSJ (*n* 21,606)^†^

	<350 g/day (*n* 15391)			≥350 g/day (*n* 6215)		
	P5	Mean	P95	P5	Mean	P95
Protein (%E)	13.8	14.7	15.5	14.6	15.5	16.3*
Total fats (%E)	22.8	26.4	29.9	23.7	27.1	30.6*
SFA (%E)	5.83	7.08	8.32	5.95	7.07	8.28*
Carbohydrates (%E)	50.0	55.0	60.3	49.3	54.3	59.3*
*n*-6 PUFA (g/MJ)	1.05	1.22	1.34	1.10	1.24	1.36*
*n*-3 PUFA (g/MJ)	0.24	0.29	0.31	0.26	0.31	0.33*
Dietary fibre (g/MJ)	1.28	1.72	2.14	1.96	2.42	2.82*
Vitamin A (μgRAE/MJ)^‡^	43.7	56.6	69.1	81.0	93.7	105.9*
Vitamin D (μg/MJ)	0.65	0.98	1.33	0.77	1.12	1.46*
Vitamin E (mg/MJ)^§^	0.67	0.79	0.89	0.87	0.99	1.09*
Vitamin K (μg/MJ)	19.6	26.8	33.7	34.2	41.3	48.3*
Vitamin B_1_ (mg/MJ)	0.10	0.11	0.11	0.11	0.12	0.13*
Vitamin B_2_ (mg/MJ)	0.12	0.15	0.17	0.13	0.16	0.18*
Niacin (mgNE/MJ)^||^	1.67	1.87	2.05	1.87	2.07	2.24*
Vitamin B_6_ (mg/MJ)	0.12	0.14	0.15	0.15	0.17	0.19*
Vitamin B_12_ (μg/MJ)	0.59	0.80	1.02	0.63	0.86	1.07
Folate (μg/MJ)	25.6	34.2	42.5	38.2	47.1	54.7*
Pantothenic acid (mg/MJ)	0.60	0.68	0.75	0.68	0.76	0.83*
Vitamin C (mg/MJ)	5.7	10.7	15.7	11.1	16.5	21.1*
Sodium, salt-equivalent (g/MJ)^¶^	1.15	1.28	1.41	1.22	1.35	1.48*
Potassium (mg/MJ)	213.4	273.2	328.2	289.9	352.8	403.2*
Calcium (mg/MJ)	44.9	60.9	75.3	57.6	74.3	87.8*
Magnesium (mg/MJ)	25.1	30.4	34.7	30.4	35.9	39.7*
Phosphorus (mg/MJ)	109.0	123.1	135.0	121.2	135.5	146.5*
Iron (mg/MJ)	0.79	0.95	1.10	0.95	1.12	1.25*
Zinc (mg/MJ)	0.96	1.00	1.03	1.03	1.08	1.10*
Copper (mg/MJ)	0.12	0.14	0.17	0.14	0.17	0.17*

%E, percentage of total daily energy intake; MUFA, monounsaturated fatty acids; NE, Niacin equivalent; NHNSJ, the National Health and Nutrition Survey in Japan; P5, the 5th percentile; P95, the 95th percentile; PUFA, polyunsaturated fatty acids; REA, retinol activity equivalents; SFA, saturated fatty acids.

† Shown as the adjusted intake of consumers based on general linear models (gamma distribution, log-link function). Covariates were adjusted for gender (men or women), age (20–29, 30–39, 40–49, 50–59, 60–69, 70–79, and ≥80 years), region (Hokkaido, Tohoku, Kanto 1 and 2, Hokuriku, Tokai, Kinki 1 and 2, Chugoku, Shikoku, and Kita- and Minami-Kyushu), weight status (<18.5, 18.5–24.9, ≥25.0 kg/m^2^, and missing), smoking experience (yes, no, and missing), and drinking habits (yes, no, or missing). Intake values are shown as the mean and 5th and 95th percentiles of the adjusted distribution in the original scale.

‡ 1 μg RAE = sum of retinol (μg) + β-carotene (μg) × 1/12 þ α-carotene (μg) × 1/12 þ β-cryptoxanthin (μg) × 1/24.

|| 1 mg NE = niacin (mg) + protein (mg)/6000.

§ Only α-tocopherol is included.

¶ Salt equivalent (g) = sodium (mg) × 2.54/1000.

* *P* < 0.001 between groups < and ≥350 g/day. *T*-test for testing the between-group difference of the least-square means obtained from the general linear models.

**Table 5.** Adherence (%) of estimated energy requirement (EER)-adjusted nutrient intake to the Dietary Reference Intakes for Japanese (2020) by groups of meeting the Japanese recommendation of vegetable intake (350 g/day), adults ≥20 years, NHNSJ 2016 (*n* 21,606)^†^

	<350 g/day (*n* 15391)	≥350 g/day (*n* 6215)
Number of nutrients meeting/DRI^‡^	*	
Larger (17–27)	24.3	58.8
Medium (13–16)	31.3	25.7
Fewer (0–12)	44.4	15.5
Nutrients with DG^§^		
Protein (%E)^||^	50.6	57.4*
Total fat (%E)^||^	49.2	51.0
SFA (%E)^¶^	52.9	52.3*
Carbohydrate (%E)^||^	58.3	60.1
Dietary fibre (g)^††^	21.7	62.5*
Sodium, salt-equivalent (g)^¶^	10.3	6.4*
Potassium (mg)^††^	27.8	65.2*
Nutrients with RDA^††^		
Vitamin A (μgRAE)	11.7	39.1*
Vitamin B_1_ (mg)	20.0	31.2*
Vitamin B_2_ (mg)	42.2	54.1*
Niacin (mgNE)	73.2	85.3*
Vitamin B_6_ (mg)	44.0	75.3*
Vitamin B_12_ (μg)	75.0	80.0*
Folate (μg)	67.9	94.7*
Vitamin C (mg)	35.4	69.5*
Calcium (mg)	24.5	39.4*
Magnesium (mg)	25.1	48.1*
Iron (mg)^‡‡^	54.6	75.4*
Zinc (mg)	41.6	51.3*
Copper (mg)	97.1	99.3*
Nutrients with AI^††^		
*n*-6 PUFA (g)	68.2	71.9*
*n*-3 PUFA (g)	60.1	63.1*
Vitamin D (μg)	34.4	41.6*
Vitamin E (mg)	57.5	76.7*
Vitamin K (μg)	60.0	87.0*
Pantothenic acid (mg)	66.6	82.3*
Phosphorus (mg)	80.9	91.1*

%E, percentage of total daily energy intake; AI, adequate intake; DG, tentative dietary goal for preventing lifestyle related disease; DRI, dietary reference intakes; EER, estimated energy requirements; NHNSJ, National Health and Nutrition Survey in Japan; PUFA, polyunsaturated fatty acids; RDA, recommended dietary allowance; REA, retinol activity equivalents; SFA, saturated fatty acids.

† Adjusted percentages of participants meeting the DRIs are shown using logistic regression. The covariates of the adjustment were gender (men or women), age (20–29, 30–39, 40–49, 50–59, 60–69, 70–79, and ≥80 years), region (Hokkaido, Tohoku, Kanto 1 and 2, Hokuriku, Tokai, Kinki 1 and 2, Chugoku, Shikoku, and Kita- and Minami-Kyushu), weight status (<18.5, 18.5–24.9, ≥25.0 kg/m^2^, and missing), smoking experience (yes, no, and missing), and drinking habits (yes, no, or missing). Except for protein, total fat, SFA, and carbohydrate, nutrient intake was adjusted to sex- and age-specific estimated energy requirement (EER) (MJ/d) at physical activity level II (i.e., normal) for comparison with DRI. EER-adjusted intake = sex- and age-specific EER (MJ/d) × nutrient intake (unit/d)/total energy intake (MJ/d). The reference values of DRI used for comparisons were DG, RDA, and AI.

‡ Based on tertiles of the number of nutrients (for each tertile, range shown in brackets) that were not adherent to the DRI.

§ Nutrients that meet the DG values.

|| Shown as the number (%) of participants with intake within the recommended range.

¶ Number (%) of participants with intake below the recommended limit. Salt equivalent (g) = sodium (mg) × 2.54/1000.

†† Shown as *n* (%) of participants with an intake above the recommended limit.

‡‡ Reference values for women refer to those set for premenopausal populations.

* *P* < 0.001 between groups < and ≥350 g/day. The Wald Chi-square test was obtained from logistic regression for the between-group difference in the percentage of consumers.

Categorising participants with vegetable intake by a 70 g increment from <70 to ≥490 g/d, linear trends were tested as significant for all nutrients for adherence to the DRI except for SFA (Table S6). Compared to the participants with vegetable intake of 280–350 g/day, participants with vegetable intake <140 g/day showed significantly smaller proportions adhered to the DRI for all nutrients, except for SFA and sodium. However, other than nutrients that have a high content in vegetables (e.g., dietary fibre, folate, potassium), for most (16 of 27) nutrients, adherences of participants from the adjacent groups (i.e., 210–280 g/day and the 350–420 g/day groups) did not significantly different from those of the reference group.

After excluding vegetable juices and fermented (or pickled) vegetables from the vegetable group, the percentage of participants who met the recommendations decreased to 24.0% (*n* = 5,181). The results of this analysis were generally consistent with the original results (data not shown). However, the percentage of consumers was not significantly different between the < 350 and ≥350 g/day groups for vegetable juices (9.0% and 10.2%, respectively; *P*-value = 0.17) or fermented (or pickled) vegetables (40.3% and 41.2%, respectively; *P*-value = 0.17). The energy-adjusted intake was significantly lower in the ≥350 g/day group than in the <350 g/day group for both vegetable juices (mean [5^th^–95^th^ percentile], 13.5 [10.3–18.5] and 18.4 [13.4–24.8] g/MJ/d, respectively) and fermented (or pickled) vegetables (mean [5^th^–95^th^ percentile], 3.1 [1.9 – 4.5] and 3.2 [1.9 – 4.9] g/MJ/d, respectively). SFA intake was no longer significantly different between the < 350 and ≥350 g/day groups (7.1 [6.0 – 8.2] and 7.1 [6.1 – 8.1] % of total energy intake/day, respectively; *P*-value = 0.003).

## Discussion

Our results revealed that participants with vegetable intake ≥350 g/day also had a larger percentage of consumers for all vegetable subgroups and other food groups (except for cereals, eggs, and condiments and seasonings) than those who consumed <350 g/day vegetables. Additionally, the energy-adjusted intake for all vegetable subgroups was higher in the ≥350 g/day group than in the <350 g/day group. For other food groups, the ≥350 g/day group had a lower energy-adjusted intake of cereals (with all subgroups), fruit juices, eggs, and beverages (with all subgroups), but a higher intake of condiments and seasonings than the <350 g/day group. Although the ≥350 g/day group had a higher intake of most nutrients and a higher percentage of participants who met the DRIs, the opposite was observed for sodium.

If potatoes and other tubers, mushrooms, and seaweeds were considered vegetables, 99.8% (99.6% if not including these food groups) of the participants consumed any vegetable on the recording/day, which is higher than that reported in the US (95%).^(31)^ In the current study, the participants had a mean intake of 283 (SD 173) g/day of vegetables and 366 (SD 210) g/day when potatoes and other tubers, mushrooms, and seaweeds were included as vegetables. The mean vegetable intake was higher than that reported in other developed countries (e.g., 183 g/day in the US^(32)^ and 152 g/day across 16 European countries^(33)^) where potatoes and other tubers were also considered vegetables. When comparing the percentage of participants who consumed the recommended vegetables, our results also revealed a higher rate (29%) than those reported in other countries (e.g., US, 10%^(7)^; UK, 8%^(8)^; and Australia, 8%^(9)^). However, the proportion of participants with vegetable intake ≥350 g/day was lower than that reported in a previous Japanese study based on the 2003 NHNSJ (35%).^(13)^

All vegetable subgroups demonstrated a higher percentage of consumers and energy-adjusted intakes in the ≥350 g/day group than in the <350 g/day group. For the vegetable juice and tertiary groups of vegetables, the percentage of consumers in the ≥350 g/day group ranged from 11.3% (fermented [or pickled] leafy vegetables) to 91.3% (other light-coloured vegetables), with a median percentage of 42.3%; the energy-adjusted intake for consumers ranged from 12.1 (fermented [or pickled] leafy vegetables) to 89.0 (vegetable juice) g/day with a median intake of 36.7 g/day. In the <350 g/day group, however, the percentages of vegetable intake ranged from 5.3% (vegetable juice) to 80.8% (other light-coloured vegetables) with a median percentage of 30.7%, and the energy-adjusted intake for consumers ranged from 10.3 (fermented [or pickled] radish or other vegetables) to 43.7 (vegetable juice) g/day with a median intake of 24.3 g/day. On average, the participants in the ≥350 g/day group consumed a greater variety of vegetables with a higher amount of each type than those in the <350 g/day group; therefore, the results may suggest that promoting the consumption of various vegetables in abundance may be necessary to meet the recommended vegetable intake. Nevertheless, vegetable juice intake was high in the <350 g/day and ≥350 g/day groups. Among the participants (*n* = 2,002) who consumed vegetable juice on the recording day, the average contribution of vegetable juice to vegetable intake was 30% (SD, 25%) (data not shown), which suggests that vegetable juice may be an important source to meet the recommended vegetable intake. There is no agreement in food-based dietary guidelines on whether vegetable juices should be classified in the vegetable category. For example, in Sweden,^(34)^ vegetable juice was excluded from the vegetable targetet because of its low fibre content, whereas in the US^(35)^ and Canada,^(36)^ it was included in the recommendations for vegetables. The association between vegetable juice intake and health outcomes is largely unknown; therefore, whether vegetable juice should be classified with vegetables requires further discussion. Further studies are needed to examine the association between vegetable juice intake and health outcomes.

For the other food groups, the energy-adjusted intake was inconsistent with the percentage of consumers. Our findings are generally in line with those of a previous study based on the 2003 NHNSJ; higher vegetable intake was associated with lower intake of wheat products, fruit juices, savoury snacks and confectionaries, and alcoholic and non-alcoholic beverages, but with higher intake of red meat, with no inter-group differences for nuts, seafood, fats, and oils.^(13)^ Our results revealed that participants with vegetable intake of ≥350 g/day had higher energy-adjusted intakes and met the DRIs for most nutrients, which is consistent with the results of previous studies showing that a higher vegetable intake was related to a higher intake of dietary fibre, potassium, calcium, magnesium, iron, vitamin C, and folate in women in the US^(37)^ and vitamins B_6_ and B_12_, C, E, and A, folic acid, and beta-carotene in European children and adolescents.^(38)^ Given that vegetables are generally high in several essential nutrients (e.g., potassium, magnesium, iron, and vitamins A, C, and K)^(1,39)^ and low in energy, a higher vegetable intake for the same daily energy intake may suggest that the diet has more energy for the intake of other foods. In our study, the ≥350 g/day group had higher percentages of consumers for most food groups, but with lower intake of fruit juices, savoury snacks and confectionaries, and beverages, which are often low in nutrient density and high in free sugars or fats.^(20,39)^ These findings suggest that participants who met the vegetable intake recommendation also had a greater variety of food groups in their diet, higher nutrient intake, and were more likely to meet the DRIs. However, adherence to the DRIs shown in this study was calculated based on a one-day dietary record and cannot be used to assess the adequacy of habitual nutrient intake.

However, our findings revealed that sodium may be a concern when promoting vegetable intake. Compared to a previous study, sodium intake was higher in our participants with vegetable intake of ≥350 g/day, which may reflect a higher intake of condiments and seasonings.^(13)^ Unlike findings from Western countries, where higher vegetable intake has been reported to have a negative^(40,41)^ or null^(42)^ relationship with sodium intake, dietary patterns with higher vegetable intake in Japan were often positively associated with higher sodium intake.^(15–17)^ This may be related to Japanese cooking practices, where salt-containing condiments and seasonings (e.g., soy sauce) are often added when preparing and preserving foods. Previous studies that have examined the relationship between vegetable intake and health outcomes (e.g., cardiovascular mortality and blood pressure) have suggested that salt added during cooking may be one of the reasons for the null findings observed between vegetable intake and these health outcomes.^(43,44)^ Future policy interventions may include promoting cooking and preserving methods with lower salt while maintaining a good taste when preparing vegetables.

This study has several limitations. First, a considerable number of participants (44.4%) refused to participate, and participants aged ≥60 years (51.6%) (compared to 30.7% in the 2010 Population Census^(45)^) may limit the generalizability of the findings.

Second, the one-day dietary record cannot be considered an accurate reflection of habitual intake because of random variations.^(46,47)^ Thus, when identifying those who meet and do not meet the recommended vegetable intake, misclassification is possible because some people may meet the recommendation based on the habitual intake but not on the day the dietary records were taken, and vice versa. For assessing the adherence to dietary recommendations for habitual intakes, future NHNSJs should consider obtaining multiple-day dietary records for at least some of the participants.^(48)^

Third, seasonal variations in dietary intake are likely.^(47,49,50)^ A study published in 2003 revealed that the total vegetable intake in autumn was the lowest (median, 282 g/day) among the four seasons (for other three seasons, median intake ranged from 286 g/day in winter to 352 g/day in summer).^(49)^ Proportion of participants who met the recommended vegetable intake may have been underestimated in this study. However, seasonal variations in vegetable purchases have become less evident because of greenhouse planting.^(51)^ More recent studies are needed to examine the seasonal variations in the intake of various food groups. Inaccurate estimation of adherence to DRIs due to seasonal variations is also likely for nutrients.^(47,50)^ For example, vitamin C intake in autumn was higher (151 mg/day) than that during the other three seasons (115 – 135 mg/day).^(50)^ As mentioned previously, future dietary assessments of the NHNSJ should include multiple days across seasons, at least in a subset of the participants.

Fourth, the misreporting of dietary intake may be related to BMI.^(52,53)^ However, we could not assess the magnitude of misreporting because of the lack of valid biomarkers. Adjusting for energy intake based on the density method may mitigate the effect of reporting errors on dietary intake.^(26,53)^

Fifth, the assessment of diet quality based on DRIs was arbitrary, as nutrients may function differently in preventing chronic diseases. However, a validated scale of diet quality for the Japanese population was not available during the analysis of this study. Thus, method based on the number of nutrients that met the DRIs was only to tentatively demonstrate the differences in the overall dietary intake between participants who met and those who did not meet the recommended vegetable intake.^(23)^ Moreover, as mentioned previously, dietary intake assessed based on one-day dietary record is prone to day-to-day variations.^(46,47)^ Thus, the objective of the current study was not to assess the adequacy of nutrient intake, but rather to show a snapshot of the overall dietary intake quality. Results of the comparison to the DRIs, therefore, cannot be interpreted or used as the assessment of dietary adequacy.

Sixth, although participants were asked to weigh food whenever possible, for some situation (e.g., eating outside the home) it was difficult to do so; estimation error in dietary intake is possible. However, trained interviewer collected and confirmed the information recorded in the dietary record by home visits to ensure the accuracy of the dietary record. Also, a previous review suggested that the differences of estimation error between estimated and weighed food intake was expected to be small.^(54)^

Seventh, as most participants in this study had vegetable intake below the recommended 350 g/day, the diet quality of participants who had a low vegetable intake (e.g., <140 g/day) may be different from those that close to the recommended intake (e.g., 280–350 g/day) (Table S6). Although our study aimed to compare the dietary intake between participants meeting and not meeting the current recommended vegetable intake, instead of addressing the population not meeting the recommended vegetable intake uniformly, public health interventions specifically targeting those with very low vegetable intake (e.g., <140 g/d) may be needed in the future.

## Conclusion

Based on the national nutrition survey in Japan, 29% of the participants met the recommendation of a vegetable intake of 350 g/day. Participants with vegetable intake ≥350 g/day also had a higher percentage of consumers and energy-adjusted intake for all vegetable subgroups. Although the percentage of consumers was also higher for other food groups in the ≥350 g/day group, the energy-adjusted intakes were lower for cereals, sugar, fruit juices, eggs, savoury snacks, and confectionaries and beverages. Participants who met the vegetable intake recommendation also had a more favourable nutrient intake profile. However, a higher sodium intake is worth noting. Higher vegetable intake in the Japanese population may indicate a diverse and generally healthy diet. It is also necessary to explore ways to increase vegetable intake without increasing the sodium intake. Future studies may need to explore individual behavioural factors related to the meeting of the recommended vegetable intake for developing more targeted plans for promoting vegetable intake.

## Supporting information

Yuan et al. supplementary material Yuan et al. supplementary material

## Supplementary material

To view supplementary material for this article, please visit https://doi.org/10.1017/jns.2024.5
