# Supplementary material for: Analysing food groups and nutrient intake in adults who met and did not meet the daily recommended vegetable intake of 350 g: the 2016 National Health and Nutrition Survey in Japan
Source: J Nutr Sci. 2024 Mar 5;13:e12. doi: 10.1017/jns.2024.5 (PMC10988146; doi:10.1017/jns.2024.5)
Supplement: Yuan et al. supplementary material [file S2048679024000053sup001.docx]

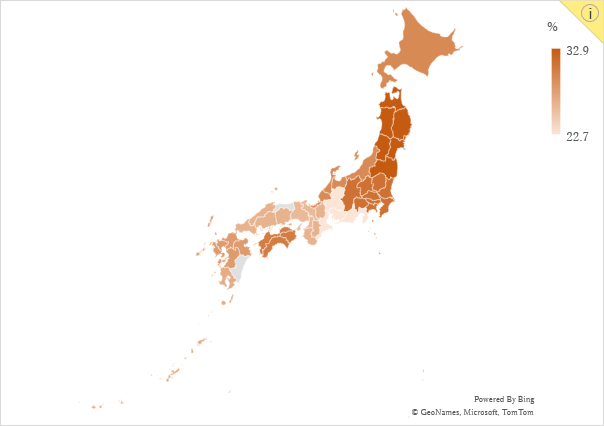


Figure S1. Percentage of participants meeting the Japanese recommendation of vegetable intake (350 g/d) by 12 regions in Japan, adults ≥20 years, the Japan National Health and Nutrition Survey, 2016. Prefectures shown in grey color are Tottori and Kumamoto, where were not included in the analysis because of Typhoon or Earthquake stricken in the survey year.

Table S1 Food groups of this study (adults >= 20 years, NHNSJ 2016, n 21606).

| **Food groups** | | |  |  |
| --- | --- | --- | --- | --- |
| **Primary** | **Secondary** | **Tertiary** |  | **Examples of food items** |
| Vegetables | Green-yellow vegetables | Tomatoes |  | Raw or canned tomatoes |
|  |  | Carrots |  | Raw, cooked, or frozen carrots |
|  |  | Spinach |  | Raw, cooked, or frozen spinach |
|  |  | Peppers |  | Raw or cooked bell (or sweet) peppers |
|  |  | Other green-yellow vegetables |  | Raw, cooked or frozen asparagus, green beans, pumpkin, kale, celery, pakchoi, parsley, leek, leaf lettuce, sunny lettuce, Brussel sprouts, broccoli |
|  | Other vegetables | Cabbages |  | Raw or cooked cabbage, red cabbage |
|  |  | Cucumbers |  | Cucumbers |
|  |  | Daikon radish |  | Raw, cooked, or dehydrated daikon radish |
|  |  | Onions |  | Raw or cooked onions |
|  |  | Chinese cabbage |  | Raw or cooked Chinese cabbage |
|  |  | Other light-colored vegetables |  | Raw, cooked, or frozen artichoke, green peas, cauliflower, zucchini, sweet corn, eggplant, garlic, beets, soybean sprouts, rhubarb, lettuce |
|  | Vegetable juices |  |  | Canned tomato (or carrot) juice, mixed vegetable juice |
|  | Fermented (or pickled) vegetables | Fermented (or pickled) leafy vegetables |  | Fermented (or pickled) green leafy vegetables |
|  |  | Fermented (or pickled) radish or other vegetables |  | Fermented (or pickled) vegetables other than green leafy vegetables |
| Potatoes and other tubers | Tubers | Sweet potatoes |  | Raw, cooked, or dehydrated sweet potatoes |
|  |  | Potatoes |  | Raw, cooked, or fried potatoes, dehydrated mashed potatoes (not included French fries) |
|  |  | Other tubers |  | Konjac, taros, yams |
|  | Starches |  |  | Tapioca, corn starch, starch noodles |
| Mushrooms |  |  |  | Raw, cooked, canned, or dehydrated fungi |
| Seaweeds |  |  |  | Raw, cooked, canned, or dehydrated seaweeds |
| Cereals | Rice products |  |  | Raw or cooked refined (or brown) rice, and rice flour and its products |
|  | Wheat products |  |  | Wheat flour, breads, sweet buns, noodles, instant noodles, pastas, and other wheat flour products such as dumpling skins |
|  | Other cereals |  |  | Buckwheat and its products (e.g., buckwheat noodles), maize and its products (e.g., popcorn and corn flake), and other grain products (e.g., oats, rye, and barley) |
| Sugar and sweeteners | |  |  | White sugar, brown sugar, maple syrup, and honey |
| Legumes | Soybeans |  |  | Dehydrated or cooked soybeans, soybean flour, tofu, natto (fermented soybeans), and other soybean products (e.g., soy milk) |
|  | Other beans |  |  | Dehydrated or cooked beans other than soybeans (e.g., red, green, black beans) and their products (e.g., fried beans and red bean paste) |
| Nuts |  |  |  | Raw, cooked, or flavored nuts (e.g., almonds, chestnuts, hazelnuts), seeds and peanuts, and their products (e.g., peanut butter) |
| Fruits | Whole fruits |  |  | Raw or canned strawberry, raw or canned citrus fruits, raw or dehydrated banana, avocado, apricots, cherry, apple, watermelon, papaya, coconuts |
|  | Jams |  |  | All fruit jams, marmalade |
|  | Fruit juices |  |  | 100% fruit juices, concentrated fruit juices, other fruit juices (e.g., 20% orange juice), nectar |
| Seafood | Unprocessed seafood | |  | Raw or cooked fish and shellfish |
|  | Processed seafood |  |  | Dehydrated, salted, canned, or soy sauce-flavored fish and shellfish, and other products (e.g., fish sausage and fish paste) |
| Meats | Red meat |  |  | Raw, cooked, or canned beef, pork, and lamb, and their products (e.g., ham and sausage) |
|  | Poultry |  |  | Raw, cooked, or canned chicken, duck, and goose |
|  | Other meats^＊^ |  |  | Offal and other meats or products (e.g., whale and frog) |
| Eggs |  |  |  | Raw, cooked, canned, fermented, or dehydrated eggs and egg products |
| Milk and dairy products^†^ |  |  |  | Milk, cheese, fermented milk (e.g., yogurt), and other dairy products (e.g., flavored and condensed milk, cream, and ice cream) |
| Fats and oils |  |  |  | Butter, margarine, plant-based oils (e.g., olive oil), animal-based fats (e.g., lard), and other fats (e.g., shortenings) |
| Savory snacks and confectionaries |  |  |  | Japanese-styled snacks and sweets, cookies, pastries, biscuits, candies, and other snacks and confectionaries (e.g., puddings, jelly, chocolate, and potato chips) |
| Beverages | Alcoholic beverages |  |  | Beers, wine, liquors |
|  | Non-alcoholic beverages | |  | Teas, coffees, coffee drinks, and soft drinks |
| Condiments and spices^‡^ |  |  |  | Sauces (e.g., Worcester sauce), soy sauce, salt, mayonnaise, miso (soybean paste), other condiments (e.g., vinegar, roux, soup stocks, tomato puree, tomato paste, ketchup, tomato sauce, chili sauce, salad dressings), and spices and others (e.g., mustard, gelatins, mixed spices, dehydrated herbs, yeast, baking powder) |
| * Combined two secondary food groups: offal, and other meats and products (e.g., whale and frog). | | | | |
| ^†^ Two secondary groups, namely "cow milk and dairy products" and "other dairies," are categorized under this group. Only the primary group was analyzed because the consumption of "other dairies" group is negligible (mean, 0.0 g/d) based on the 2016 National Health and Nutrition Survey in Japan. | | | | |
| ^‡^ Two secondary groups, namely "condiments" and "seasonings, spices, and others," are categorized under this group. Only the primary group was analyzed because the consumption of "seasonings, spices, and others" group is negligible (mean, 0.0 g/d) based on the 2016 National Health and Nutrition Survey in Japan. | | | | |

Table S2. Reference values of Dietary Reference Intakes of Japanese (2020) used in this study (adults ≥ 20 years, NHNSJ 2016, n 21606).

|  |  | Men | | | | |  | Women | | | | |
| --- | --- | --- | --- | --- | --- | --- | --- | --- | --- | --- | --- | --- |
| Age groups | | 18-29 | 30-49 | 50-64 | 65-74 | 75+ |  | 18-29 | 30-49 | 50-64 | 65-74 | 75+ |
| EER^*^ | | 2650 | 2700 | 2600 | 2400 | 2100 |  | 2000 | 2050 | 1950 | 1850 | 1650 |
| Nutrients with DG | |  |  |  |  |  |  |  |  |  |  |  |
|  | Protein (%E) | 13-20 | 13-20 | 14-20 | 15-20 | 15-20 |  | 13-20 | 13-20 | 14-20 | 15-20 | 15-20 |
|  | Total fat (%E) | 20-30 | 20-30 | 20-30 | 20-30 | 20-30 |  | 20-30 | 20-30 | 20-30 | 20-30 | 20-30 |
|  | SFA | <=7 | <=7 | <=7 | <=7 | <=7 |  | <=7 | <=7 | <=7 | <=7 | <=7 |
|  | Carbohydrate (%E) | 50-65 | 50-65 | <=7 | 50-65 | 50-65 |  | 50-65 | 50-65 | 50-65 | 50-65 | 50-65 |
|  | Dietary fiber (g) | >=21 | >=21 | >=21 | >=20 | >=20 |  | >=18 | >=18 | >=18 | >=17 | >=17 |
|  | Salt-equivalent (g) | <=7.5 | <=7.5 | <=7.5 | <=7.5 | <=7.5 |  | <=6.5 | <=6.5 | <=6.5 | <=6.5 | <=6.5 |
|  | Potassium (mg) | >=3000 | >=3000 | >=3000 | >=3000 | >=3000 |  | >=2600 | >=2600 | >=2600 | >=2600 | >=2600 |
| Nutrients with RDA | |  |  |  |  |  |  |  |  |  |  |  |
|  | Vitamin A (μgRAE) | 850 | 900 | 900 | 850 | 800 |  | 650 | 700 | 700 | 700 | 650 |
|  | Vitamin B_1_ (mg) | 1.4 | 1.4 | 1.3 | 1.3 | 1.2 |  | 1.1 | 1.1 | 1.1 | 1.1 | 0.9 |
|  | Vitamin B_2_ (mg) | 1.6 | 1.6 | 1.5 | 1.5 | 1.3 |  | 1.2 | 1.2 | 1.2 | 1.2 | 1 |
|  | Niacin (mgNE) | 15 | 15 | 14 | 14 | 13 |  | 11 | 12 | 11 | 11 | 11 |
|  | Vitamin B_6_ (mg) | 1.4 | 1.4 | 1.4 | 1.4 | 1.4 |  | 1.1 | 1.1 | 1.1 | 1.1 | 1.1 |
|  | Vitamin B_12_ (μg) | 2.4 | 2.4 | 2.4 | 2.4 | 2.4 |  | 2.4 | 2.4 | 2.4 | 2.4 | 2.4 |
|  | Folate (μg) | 240 | 240 | 240 | 240 | 240 |  | 240 | 240 | 240 | 240 | 240 |
|  | Vitamin C (mg) | 100 | 100 | 100 | 100 | 100 |  | 100 | 100 | 100 | 100 | 100 |
|  | Calcium (mg) | 800 | 750 | 750 | 750 | 700 |  | 650 | 650 | 650 | 650 | 600 |
|  | Magnesium (mg) | 340 | 370 | 370 | 350 | 320 |  | 270 | 290 | 290 | 280 | 260 |
|  | Iron (mg)^†^ | 7.5 | 7.5 | 7.5 | 7.5 | 7 |  | 10.5 | 10.5 | 11 | 6 | 6 |
|  | Zinc (mg) | 11 | 11 | 11 | 11 | 10 |  | 8 | 8 | 8 | 8 | 8 |
|  | Copper (mg) | 0.9 | 0.9 | 0.9 | 0.9 | 0.9 |  | 0.7 | 0.7 | 0.7 | 0.7 | 0.7 |
| Nutrients with AI | |  |  |  |  |  |  |  |  |  |  |  |
|  | n-6 PUFA (g) | 11 | 10 | 10 | 9 | 8 |  | 8 | 8 | 8 | 8 | 7 |
|  | n-3 PUFA (g) | 2 | 2 | 2.2 | 2.2 | 2.1 |  | 1.6 | 1.6 | 1.9 | 2 | 1.8 |
|  | Vitamin D (μg) | 8.5 | 8.5 | 8.5 | 8.5 | 8.5 |  | 8.5 | 8.5 | 8.5 | 8.5 | 8.5 |
|  | Vitamin E (mg) | 6 | 6 | 7 | 7 | 6.5 |  | 5 | 5 | 6 | 6.5 | 6.5 |
|  | Vitamin K (μg) | 150 | 150 | 150 | 150 | 150 |  | 150 | 150 | 150 | 150 | 150 |
|  | Pantothenic acid (mg) | 5 | 5 | 6 | 6 | 6 |  | 5 | 5 | 5 | 5 | 5 |
|  | Phosphorus (mg) | 1000 | 1000 | 1000 | 1000 | 1000 |  | 800 | 800 | 800 | 800 | 800 |

AI, adequate intake; DG, tentative dietary goal for preventing life-style related disease; DRI, dietary reference intakes; EER, estimated energy requirements; PUFA, polyunsaturated fatty acids; RDA, recommended dietary allowance; REA, retinol activity equivalents; SFA, saturated fatty acids.

^*^ EER for physical activity level at II (i.e., normal).

^†^ Reference values are for pre-menopausal populations.

Table S3 Crude intakes for vegetables, potatoes and tubers, mushrooms, and seaweeds in all participants and by groups of meeting the Japanese recommendation of vegetable intake (350 g/d), adults ≥ 20 years, 2016 NHNSJ (n 21606)^*^

|  |  |  | All (n 21606) | | | | |  | < 350 g/d (n 15391) | | | | |  | ≥ 350 g/d (n 6215) | | | | |
| --- | --- | --- | --- | --- | --- | --- | --- | --- | --- | --- | --- | --- | --- | --- | --- | --- | --- | --- | --- |
|  |  |  | Mean | SD | Median | Q1 | Q3 |  | Mean | SD | Median | Q1 | Q3 |  | Mean | SD | Median | Q1 | Q3 |
| Vegetables | | | 282.7 | 172.9 | 254.6 | 159.0 | 372.0 |  | 196.2 | 86.6 | 198.5 | 129.5 | 267.5 |  | 495.4 | 146.0 | 452.3 | 394.6 | 549.6 |
|  | Green-yellow vegetables | | 95.8 | 82.6 | 74.5 | 36.0 | 133.0 |  | 67.7 | 51.5 | 56.3 | 27.9 | 97.0 |  | 160.1 | 102.1 | 145.0 | 85.0 | 214.7 |
|  |  | Tomatoes | 55.6 | 52.7 | 40.0 | 20.0 | 72.8 |  | 40.9 | 33.9 | 30.0 | 20.0 | 53.0 |  | 75.7 | 65.7 | 55.4 | 30.0 | 100.0 |
|  |  | Carrots | 29.1 | 27.5 | 21.6 | 10.4 | 38.8 |  | 22.8 | 18.6 | 18.0 | 10.0 | 30.8 |  | 41.4 | 36.5 | 33.0 | 18.0 | 53.7 |
|  |  | Spinach | 55.1 | 44.3 | 45.0 | 25.0 | 71.5 |  | 47.0 | 34.5 | 42.0 | 21.0 | 65.0 |  | 69.1 | 54.9 | 57.0 | 33.3 | 90.0 |
|  |  | Bell peppers | 24.5 | 23.5 | 17.3 | 10.0 | 31.2 |  | 20.8 | 20.3 | 15.0 | 8.0 | 26.0 |  | 29.9 | 26.6 | 22.0 | 12.0 | 39.0 |
|  |  | Other green-yellow vegetables | 52.8 | 56.6 | 35.4 | 12.0 | 73.7 |  | 39.2 | 39.5 | 27.9 | 10.0 | 55.3 |  | 79.6 | 73.2 | 60.0 | 25.0 | 114.8 |
|  | Other vegetables | | 173.7 | 120.4 | 150.0 | 89.5 | 231.0 |  | 124.7 | 69.1 | 117.6 | 71.0 | 171.5 |  | 292.3 | 134.6 | 278.4 | 203.8 | 356.1 |
|  |  | Cabbages | 63.7 | 55.9 | 50.0 | 25.0 | 85.0 |  | 49.7 | 39.0 | 40.0 | 20.0 | 66.7 |  | 88.7 | 70.7 | 71.4 | 40.0 | 120.0 |
|  |  | Cucumbers | 31.7 | 29.8 | 24.5 | 12.3 | 41.0 |  | 25.9 | 22.4 | 20.0 | 10.0 | 33.3 |  | 42.5 | 37.8 | 30.5 | 19.6 | 51.9 |
|  |  | Daikon radish | 69.5 | 65.2 | 50.0 | 26.7 | 90.0 |  | 54.4 | 44.3 | 40.0 | 21.4 | 74.5 |  | 94.1 | 83.6 | 70.0 | 36.0 | 123.9 |
|  |  | Onions | 51.7 | 45.0 | 40.0 | 20.0 | 70.5 |  | 42.2 | 33.5 | 34.5 | 17.1 | 58.8 |  | 71.2 | 57.6 | 57.0 | 30.0 | 99.0 |
|  |  | Chinese cabbage | 73.3 | 68.9 | 52.4 | 25.0 | 100.0 |  | 55.1 | 47.1 | 43.3 | 20.0 | 78.3 |  | 102.3 | 86.1 | 80.0 | 37.5 | 143.7 |
|  |  | Other vegetables | 59.2 | 59.5 | 42.0 | 18.1 | 82.0 |  | 44.1 | 39.9 | 33.0 | 13.9 | 62.0 |  | 92.5 | 78.8 | 75.0 | 35.5 | 125.0 |
|  | Vegetable juices | | 129.5 | 123.3 | 150.0 | 5.0 | 200.0 |  | 70.4 | 82.9 | 7.8 | 3.0 | 150.0 |  | 170.7 | 130.0 | 190.0 | 100.0 | 206.0 |
|  | Fermented (or pickled) vegetables | | 24.9 | 27.3 | 15.0 | 10.0 | 30.0 |  | 21.7 | 21.6 | 15.0 | 10.0 | 30.0 |  | 31.9 | 35.6 | 20.0 | 10.0 | 40.0 |
|  |  | Fermented (or pickled) leafy vegetables | 31.4 | 29.4 | 20.0 | 10.0 | 40.0 |  | 27.7 | 24.5 | 20.0 | 10.0 | 35.0 |  | 38.0 | 35.5 | 25.0 | 15.0 | 50.0 |
|  |  | Fermented (or pickled) radish or other vegetables | 20.6 | 22.8 | 13.0 | 8.0 | 25.0 |  | 18.2 | 18.1 | 11.3 | 7.5 | 22.1 |  | 26.0 | 30.1 | 15.0 | 10.0 | 30.0 |
| Potatoes and other tubers | | | 33.2 | 33.0 | 23.0 | 11.0 | 45.0 |  | 29.2 | 29.5 | 20.0 | 10.0 | 40.0 |  | 41.2 | 37.9 | 30.0 | 15.0 | 54.0 |
|  | Tubers | | 83.8 | 71.0 | 65.0 | 35.0 | 109.7 |  | 77.4 | 64.9 | 60.0 | 33.0 | 100.0 |  | 98.1 | 81.2 | 78.0 | 40.9 | 131.4 |
|  |  | Sweet potatoes | 60.1 | 57.3 | 45.0 | 24.7 | 78.5 |  | 57.7 | 56.3 | 40.0 | 22.9 | 72.3 |  | 64.9 | 59.0 | 50.0 | 27.0 | 90.0 |
|  |  | Potatoes | 68.8 | 53.5 | 54.5 | 32.9 | 90.0 |  | 63.9 | 47.8 | 52.5 | 30.0 | 82.0 |  | 79.8 | 63.0 | 60.5 | 39.0 | 102.0 |
|  |  | Other tubers | 60.7 | 55.3 | 45.0 | 23.0 | 80.7 |  | 56.7 | 51.5 | 40.0 | 20.5 | 76.0 |  | 69.1 | 61.6 | 50.0 | 26.7 | 95.3 |
|  | Starches | | 8.3 | 15.3 | 3.5 | 1.6 | 7.3 |  | 7.4 | 13.0 | 3.4 | 1.6 | 7.0 |  | 10.4 | 19.8 | 4.0 | 1.9 | 8.8 |
| Mushrooms | | | 20.2 | 27.7 | 10.8 | 4.0 | 25.8 |  | 18.8 | 25.5 | 10.3 | 3.2 | 23.9 |  | 23.3 | 31.9 | 13.0 | 5.0 | 31.5 |
| Seaweeds | | | 76.0 | 71.5 | 59.4 | 26.8 | 102.0 |  | 69.6 | 65.4 | 54.0 | 25.0 | 95.0 |  | 90.9 | 81.9 | 72.0 | 32.5 | 124.8 |

NHNSJ, the National Health and Nutrition Survey in Japan; SD, standard deviation; Q1, 25th percentile; Q3, 75th percentile.

^*^ Intakes were analyzed for consumers (>= 0 g/d) only.

Table S4 Crude intakes for other food groups in all participants and by groups of meeting the Japanese recommendation of vegetable intake (350 g/d), adults ≥20 years, 2016 NHNSJ (n 21606) ^*^

|  |  | All (n 21606) | | | | |  | < 350 g/d (n 15391) | | | | |  | ≥ 350 g/d (n 6215) | | | | |
| --- | --- | --- | --- | --- | --- | --- | --- | --- | --- | --- | --- | --- | --- | --- | --- | --- | --- | --- |
|  |  | Mean | SD | Median | Q1 | Q3 |  | Mean | Std | Median | Q1 | Q3 |  | Mean | Std | Median | Q1 | Q3 |
| Cereals | | 429.4 | 174.9 | 413.5 | 306.0 | 527.8 |  | 427.9 | 174.0 | 412.0 | 305.0 | 527.4 |  | 433.1 | 177.0 | 416.7 | 310.0 | 528.0 |
|  | Rice products | 332.1 | 171.8 | 300.0 | 200.0 | 424.0 |  | 329.4 | 169.9 | 300.0 | 200.0 | 420.0 |  | 338.6 | 176.2 | 307.5 | 200.0 | 440.0 |
|  | Wheat products | 119.8 | 104.3 | 88.0 | 47.0 | 180.0 |  | 121.2 | 104.8 | 90.0 | 48.3 | 180.0 |  | 116.0 | 102.9 | 83.2 | 45.0 | 173.7 |
|  | Other cereals | 85.0 | 94.6 | 40.0 | 18.0 | 144.4 |  | 87.3 | 93.1 | 42.1 | 19.5 | 150.0 |  | 80.7 | 97.1 | 40.0 | 15.0 | 120.0 |
| Sugar and sweeteners | | 8.7 | 8.7 | 6.0 | 3.0 | 11.5 |  | 8.4 | 8.5 | 6.0 | 3.0 | 11.0 |  | 9.6 | 9.1 | 7.0 | 3.2 | 13.0 |
| Legumes | | 86.3 | 78.0 | 63.9 | 32.2 | 117.5 |  | 80.3 | 74.5 | 58.5 | 30.0 | 106.7 |  | 99.6 | 83.7 | 76.0 | 40.0 | 135.0 |
|  | Soybeans | 85.5 | 77.3 | 62.5 | 32.0 | 115.0 |  | 79.6 | 74.0 | 57.6 | 30.0 | 105.0 |  | 98.4 | 82.7 | 75.2 | 40.0 | 134.0 |
|  | Other beans | 29.8 | 34.1 | 20.0 | 10.0 | 39.0 |  | 28.5 | 31.2 | 20.0 | 10.0 | 35.0 |  | 32.4 | 39.2 | 21.4 | 10.0 | 40.0 |
| Nuts |  | 9.0 | 16.6 | 4.0 | 1.5 | 10.0 |  | 8.7 | 17.4 | 3.6 | 1.5 | 9.3 |  | 9.5 | 15.1 | 4.5 | 1.8 | 10.9 |
| Fruits | | 162.7 | 131.2 | 128.0 | 75.0 | 219.5 |  | 150.7 | 123.0 | 119.0 | 66.5 | 201.0 |  | 186.7 | 143.1 | 153.1 | 88.0 | 253.0 |
|  | Whole fruits | 161.8 | 120.9 | 128.0 | 80.0 | 213.0 |  | 150.0 | 111.9 | 119.0 | 75.0 | 199.5 |  | 184.5 | 133.7 | 150.0 | 88.8 | 248.3 |
|  | Jams | 13.8 | 10.5 | 10.0 | 7.0 | 20.0 |  | 13.4 | 10.0 | 10.0 | 7.0 | 20.0 |  | 14.6 | 11.3 | 10.5 | 7.0 | 20.0 |
|  | Fruit juices | 70.2 | 119.3 | 9.0 | 3.0 | 102.5 |  | 78.9 | 124.9 | 9.0 | 3.0 | 150.0 |  | 55.5 | 107.8 | 8.8 | 3.3 | 50.0 |
| Seafood | | 89.8 | 70.2 | 79.0 | 38.1 | 122.5 |  | 85.1 | 67.6 | 73.8 | 34.6 | 117.5 |  | 100.7 | 74.8 | 89.7 | 47.0 | 136.3 |
|  | Unprocessed seafood | 83.8 | 61.5 | 73.5 | 40.0 | 106.7 |  | 79.9 | 60.0 | 70.0 | 39.0 | 102.0 |  | 92.7 | 63.8 | 80.0 | 50.0 | 118.0 |
|  | Processed seafood | 45.8 | 46.2 | 30.0 | 10.3 | 70.0 |  | 43.8 | 44.7 | 30.0 | 10.0 | 66.0 |  | 50.1 | 49.0 | 35.7 | 12.0 | 75.0 |
| Meats | | 100.9 | 72.8 | 85.2 | 49.8 | 135.7 |  | 94.8 | 69.0 | 80.0 | 45.0 | 128.3 |  | 115.4 | 79.5 | 100.0 | 60.0 | 151.8 |
|  | Red meat | 77.9 | 59.7 | 64.0 | 33.7 | 105.0 |  | 72.6 | 55.9 | 60.0 | 30.0 | 100.0 |  | 90.6 | 66.3 | 76.7 | 41.5 | 120.3 |
|  | Poultry | 71.3 | 58.8 | 55.0 | 30.0 | 99.3 |  | 68.7 | 56.6 | 50.0 | 28.1 | 94.0 |  | 77.5 | 63.3 | 60.0 | 32.8 | 100.0 |
|  | Other meats ^†^ | 66.1 | 56.0 | 50.0 | 30.0 | 85.0 |  | 64.2 | 50.8 | 50.0 | 30.0 | 84.5 |  | 69.9 | 65.0 | 50.0 | 30.0 | 88.8 |
| Eggs | | 48.0 | 33.1 | 44.0 | 23.6 | 62.0 |  | 47.0 | 33.2 | 44.0 | 21.5 | 61.0 |  | 50.6 | 32.6 | 47.2 | 27.5 | 64.5 |
| Milk and dairy products | | 154.3 | 134.4 | 124.0 | 50.0 | 218.0 |  | 147.9 | 133.8 | 114.0 | 38.6 | 210.7 |  | 168.7 | 134.8 | 150.0 | 70.0 | 241.0 |
| Fats and oils | | 11.7 | 9.4 | 9.6 | 5.0 | 16.0 |  | 11.4 | 9.1 | 9.3 | 4.9 | 15.6 |  | 12.6 | 10.0 | 10.0 | 5.2 | 17.0 |
| Savory snacks and   confectionaries | | 56.7 | 51.0 | 42.5 | 20.0 | 80.0 |  | 58.4 | 52.3 | 45.0 | 20.0 | 80.0 |  | 52.9 | 47.8 | 40.0 | 20.0 | 73.0 |
| Beverages | | 698.7 | 485.5 | 602.5 | 357.5 | 935.2 |  | 689.8 | 486.9 | 600.0 | 353.0 | 915.0 |  | 720.4 | 481.4 | 631.0 | 371.5 | 965.3 |
|  | Alcoholic beverages | 242.0 | 357.9 | 22.3 | 5.0 | 358.5 |  | 251.4 | 374.6 | 20.0 | 5.0 | 360.0 |  | 221.6 | 317.8 | 25.6 | 6.3 | 354.4 |
|  | Non-alcoholic beverages | 602.0 | 398.9 | 530.0 | 304.0 | 800.0 |  | 592.7 | 394.3 | 514.0 | 302.0 | 782.0 |  | 624.5 | 409.2 | 551.0 | 330.0 | 805.0 |
| Condiments and seasonings | | 97.0 | 98.2 | 63.2 | 41.0 | 107.5 |  | 89.8 | 92.0 | 57.4 | 36.8 | 97.7 |  | 114.8 | 109.8 | 77.6 | 53.4 | 125.8 |

NHNSJ, the National Health and Nutrition Survey in Japan; SD, standard deviation; Q1, 25th percentile; Q3, 75th percentile.

^*^ Intakes were analyzed for consumers (>= 0 g/d) only.

^†^ Included offal and other meat (e.g., frog).

Table S5 Crude intakes for nutrients in all participants and by groups of meeting the Japanese recommendation of vegetable intake (350 g/d), adults ≥20 years, 2016 NHNSJ (n 21606)

|  | All (n 21606) | | | | |  | < 350 g/d (n 15391) | | | | |  | >= 350 g/d (n 6215) | | | | |
| --- | --- | --- | --- | --- | --- | --- | --- | --- | --- | --- | --- | --- | --- | --- | --- | --- | --- |
|  | Mean | SD | Median | Q1 | Q3 |  | Mean | SD | Median | Q1 | Q3 |  | Mean | SD | Median | Q1 | Q3 |
| Energy (MJ) | 448 | 132 | 435 | 359 | 524 |  | 427 | 125 | 415 | 343 | 498 |  | 502 | 132 | 487 | 409 | 578 |
| Protein (g) | 69.4 | 23.2 | 67.1 | 53.6 | 82.5 |  | 64.9 | 21.3 | 63.0 | 50.3 | 77.3 |  | 80.7 | 23.8 | 78.0 | 64.4 | 93.5 |
| Total fat (g) | 55.9 | 24.0 | 52.9 | 39.1 | 69.1 |  | 52.8 | 22.7 | 50.1 | 36.7 | 65.4 |  | 63.6 | 25.2 | 60.4 | 46.2 | 77.4 |
| SFA (g) | 14.9 | 7.4 | 13.8 | 9.6 | 18.8 |  | 14.2 | 7.2 | 13.2 | 9.1 | 18.0 |  | 16.6 | 7.7 | 15.5 | 11.2 | 20.7 |
| n-6 PUFA (g) | 9.6 | 4.8 | 8.8 | 6.2 | 12.2 |  | 9.1 | 4.6 | 8.3 | 5.8 | 11.5 |  | 10.9 | 5.1 | 10.2 | 7.3 | 13.6 |
| n-3 PUFA (g) | 2.3 | 1.5 | 2.0 | 1.2 | 3.0 |  | 2.1 | 1.4 | 1.8 | 1.1 | 2.8 |  | 2.7 | 1.6 | 2.3 | 1.5 | 3.4 |
| Carbohydrate (g) | 255.2 | 80.6 | 248.3 | 201.1 | 301.1 |  | 244.0 | 77.5 | 237.4 | 192.2 | 288.5 |  | 283.0 | 81.5 | 274.6 | 227.4 | 328.3 |
| Dietary fiber (g) | 14.8 | 6.7 | 13.8 | 10.1 | 18.3 |  | 12.5 | 5.0 | 11.9 | 8.9 | 15.3 |  | 20.6 | 6.9 | 19.4 | 15.9 | 24.0 |
| Vitamin A (μgRAE)^*^ | 523.4 | 771.2 | 395.5 | 242.3 | 608.6 |  | 413.3 | 710.8 | 328.9 | 205.2 | 483.4 |  | 795.9 | 844.0 | 638.7 | 437.7 | 923.4 |
| Vitamin D (μg) | 7.9 | 8.9 | 4.4 | 1.8 | 11.5 |  | 7.2 | 8.3 | 3.8 | 1.6 | 10.2 |  | 9.7 | 9.9 | 6.2 | 2.5 | 13.8 |
| Vitamin E (mg)^†^ | 6.6 | 3.3 | 6.0 | 4.3 | 8.3 |  | 5.8 | 2.8 | 5.4 | 3.8 | 7.3 |  | 8.5 | 3.6 | 7.9 | 5.9 | 10.3 |
| Vitamin K (μg) | 237.8 | 181.4 | 184.1 | 104.4 | 333.7 |  | 193.6 | 149.2 | 147.4 | 86.5 | 264.1 |  | 347.5 | 205.9 | 305.1 | 192.6 | 460.5 |
| Vitamin B_1_ (mg) | 0.9 | 0.4 | 0.8 | 0.6 | 1.0 |  | 0.8 | 0.4 | 0.7 | 0.5 | 0.9 |  | 1.0 | 0.4 | 1.0 | 0.8 | 1.2 |
| Vitamin B_2_ (mg) | 1.2 | 0.5 | 1.1 | 0.8 | 1.4 |  | 1.1 | 0.5 | 1.0 | 0.7 | 1.3 |  | 1.4 | 0.5 | 1.3 | 1.0 | 1.6 |
| Niacin (mgNE)^‡^ | 15.0 | 7.1 | 13.8 | 10.2 | 18.4 |  | 13.8 | 6.7 | 12.7 | 9.3 | 16.9 |  | 17.9 | 7.2 | 16.6 | 12.9 | 21.7 |
| Vitamin B_6_ (mg) | 1.1 | 0.5 | 1.1 | 0.8 | 1.4 |  | 1.0 | 0.4 | 1.0 | 0.7 | 1.2 |  | 1.5 | 0.5 | 1.4 | 1.1 | 1.7 |
| Vitamin B_12_ (μg) | 6.4 | 6.8 | 4.1 | 2.1 | 8.1 |  | 5.9 | 6.5 | 3.8 | 1.9 | 7.4 |  | 7.5 | 7.4 | 5.1 | 2.6 | 9.6 |
| Folate (μg) | 291.7 | 149.8 | 268.3 | 195.7 | 357.6 |  | 247.8 | 124.9 | 234.4 | 172.8 | 302.5 |  | 400.2 | 151.3 | 374.3 | 303.1 | 465.5 |
| Pantothenic acid (mg) | 5.5 | 2.0 | 5.2 | 4.1 | 6.6 |  | 5.0 | 1.8 | 4.8 | 3.8 | 6.0 |  | 6.6 | 2.0 | 6.3 | 5.2 | 7.7 |
| Vitamin C (mg) | 95.9 | 71.5 | 78.3 | 45.7 | 126.6 |  | 77.9 | 59.6 | 63.0 | 37.3 | 101.2 |  | 140.5 | 78.6 | 122.8 | 84.9 | 177.8 |
| Sodium,  salt-equivalent (g)^§^ | 9.9 | 3.9 | 9.5 | 7.2 | 12.1 |  | 9.3 | 3.6 | 8.9 | 6.7 | 11.3 |  | 11.6 | 4.3 | 11.0 | 8.6 | 13.9 |
| Potassium (mg) | 2290.7 | 910.8 | 2179.8 | 1652.4 | 2796.7 |  | 1996.1 | 727.4 | 1928.6 | 1485.8 | 2424.0 |  | 3020.3 | 909.0 | 2890.6 | 2385.3 | 3493.6 |
| Calcium (mg) | 500.1 | 259.2 | 456.9 | 310.2 | 643.0 |  | 445.6 | 232.6 | 403.7 | 274.6 | 572.1 |  | 635.1 | 272.0 | 593.8 | 438.8 | 781.8 |
| Magnesium (mg) | 247.0 | 92.7 | 235.2 | 182.6 | 297.7 |  | 222.5 | 79.1 | 213.7 | 167.5 | 267.8 |  | 307.7 | 95.9 | 293.3 | 241.5 | 357.7 |
| Phosphorus (mg) | 984.3 | 337.7 | 954.1 | 750.7 | 1177.3 |  | 908.2 | 304.9 | 884.5 | 696.7 | 1087.0 |  | 1172.8 | 341.0 | 1132.0 | 938.7 | 1358.0 |
| Iron (mg) | 7.7 | 3.1 | 7.3 | 5.6 | 9.4 |  | 7.0 | 2.7 | 6.7 | 5.1 | 8.4 |  | 9.6 | 3.3 | 9.2 | 7.4 | 11.3 |
| Zinc (mg) | 8.0 | 2.8 | 7.6 | 6.1 | 9.4 |  | 7.4 | 2.6 | 7.1 | 5.7 | 8.8 |  | 9.3 | 2.9 | 8.9 | 7.4 | 10.7 |
| Copper (mg) | 1.1 | 0.4 | 1.1 | 0.9 | 1.4 |  | 1.1 | 0.4 | 1.0 | 0.8 | 1.3 |  | 1.4 | 0.4 | 1.3 | 1.1 | 1.6 |

MUFA, monounsaturated fatty acids; NE, Niacin equivalent; NHNSJ, the National Health and Nutrition Survey in Japan; PUFA, polyunsaturated fatty acids; REA, retinol activity equivalents; SFA, saturated fatty acids.

^*^ 1 μg RAE = sum of retinol (μg) + β-carotene (μg) × 1/12 þ α-carotene (μg) × 1/12 þ β-cryptoxanthin (μg) × 1/24.

^†^ 1 mg NE = niacin (mg) + protein (mg)/6000.

^‡^ Only α-tocopherol was included.

^§^ Salt equivalent (g) = sodium (mg) × 2.54/1000.

Table S6. Adherence (%) of estimated energy requirement (EER)-adjusted nutrient intake to the Dietary Reference Intakes for Japanese (2020) by vegetable intake categories, adults >= 20 years, NHNSJ 2016 (n 21606).

|  |  | vegetable intake categories (g/d) | | | | | | | | | | | | | | | | | |
| --- | --- | --- | --- | --- | --- | --- | --- | --- | --- | --- | --- | --- | --- | --- | --- | --- | --- | --- | --- |
|  |  | <70 | | 70-<140 | | 140-<210 | | 210-<280 | | **280-<350** | | 350-<420 | | 420-<490 | | >=490 | |  |  |
|  |  | N | % | N | % | N | % | N | % | **N** | **%** | N | % | N | % | N | % | *P*_trend_* |  |
| All |  | 1408 | 100.0 | 3052 | 100.0 | 3904 | 100.0 | 3867 | 100.0 | **3158** | **100.0** | 2295 | 100.0 | 1559 | 100.0 | 2363 | 100.0 |  |  |
| Number of nutrients meeting DRI† | | |  |  |  |  |  |  |  |  |  |  |  |  |  |  |  |  |  |
|  | Larger (17-27) | 97 | 6.9* | 379 | 12.4* | 820 | 21* | 1187 | 30.7* | **1256** | **39.8** | 1163 | 50.7* | 842 | 54* | 1648 | 69.7* | <.0001 |  |
| Nutrients with DG § | |  |  |  |  |  |  |  |  |  |  |  |  |  |  |  |  |  |  |
|  | Protein (%E) \|\| | 583 | 41.4* | 1411 | 46.2* | 1986 | 50.9* | 2067 | 53.5 | **1742** | **55.2** | 1332 | 58.0 | 858 | 55.0 | 1377 | 58.3* | <.0001 |  |
|  | Total fat (%E) \|\| | 623 | 44.2* | 1431 | 46.9 | 1932 | 49.5 | 2001 | 51.7 | **1586** | **50.2** | 1195 | 52.1 | 767 | 49.2 | 1207 | 51.1 | <.0001 |  |
|  | SFA (%E) ¶ | 829 | 58.9* | 1638 | 53.7 | 2020 | 51.7 | 1999 | 51.7 | **1658** | **52.5** | 1168 | 50.9 | 792 | 50.8 | 1290 | 54.6 | 0.06 |  |
|  | Carbohydrate (%E) \|\| | 775 | 55.0 | 1725 | 56.5 | 2319 | 59.4 | 2279 | 58.9 | **1876** | **59.4** | 1382 | 60.2 | 926 | 59.4 | 1429 | 60.5 | 0.0002 |  |
|  | Dietary fiber (g) †† | 78 | 5.5* | 324 | 10.6* | 668 | 17.1* | 1073 | 27.7* | **1201** | **38.0** | 1180 | 51.4* | 900 | 57.7* | 1807 | 76.5* | <.0001 |  |
|  | Sodium, salt-equivalent (g) ¶ | 285 | 20.2* | 397 | 13* | 375 | 9.6 | 286 | 7.4 | **236** | **7.5** | 148 | 6.4 | 99 | 6.4 | 152 | 6.4 | <.0001 |  |
|  | Potassium (mg) †† | 175 | 12.4* | 495 | 16.2* | 934 | 23.9* | 1271 | 32.9* | **1400** | **44.3** | 1291 | 56.3* | 942 | 60.4* | 1819 | 77* | <.0001 |  |
| Nutrients with RDA †† | |  |  |  |  |  |  |  |  |  |  |  |  |  |  |  |  |  |  |
|  | Vitamin A (μgRAE) | 41 | 2.9* | 113 | 3.7* | 357 | 9.1* | 561 | 14.5* | **732** | **23.2** | 690 | 30.1* | 565 | 36.2* | 1179 | 49.9* | <.0001 |  |
|  | Vitamin B1 (mg) | 237 | 16.8* | 520 | 17* | 722 | 18.5* | 843 | 21.8 | **748** | **23.7** | 597 | 26.0 | 449 | 28.8* | 896 | 37.9* | <.0001 |  |
|  | Vitamin B2 (mg) | 557 | 39.6* | 1163 | 38.1* | 1567 | 40.1* | 1729 | 44.7 | **1474** | **46.7** | 1190 | 51.9 | 826 | 53* | 1349 | 57.1* | <.0001 |  |
|  | Niacin (mgNE) | 872 | 61.9* | 2067 | 67.7* | 2854 | 73.1* | 2949 | 76.3* | **2517** | **79.7** | 1930 | 84.1* | 1298 | 83.3 | 2077 | 87.9* | <.0001 |  |
|  | Vitamin B6 (mg) | 353 | 25.1 | 961 | 31.5* | 1669 | 42.8* | 1926 | 49.8* | **1867** | **59.1** | 1609 | 70.1* | 1119 | 71.8* | 1954 | 82.7* | <.0001 |  |
|  | Vitamin B12 (μg) | 962 | 68.3 | 2178 | 71.4* | 2938 | 75.3* | 3014 | 77.9 | **2454** | **77.7** | 1833 | 79.9 | 1238 | 79.4 | 1901 | 80.4 | <.0001 |  |
|  | Folate (μg) | 522 | 37.1* | 1519 | 49.8* | 2562 | 65.6* | 3088 | 79.9* | **2765** | **87.6** | 2112 | 92* | 1466 | 94* | 2310 | 97.8* | <.0001 |  |
|  | Vitamin C (mg) | 251 | 17.8* | 692 | 22.7* | 1236 | 31.7* | 1604 | 41.5* | **1663** | **52.7** | 1395 | 60.8* | 1027 | 65.9* | 1896 | 80.2* | <.0001 |  |
|  | Calcium (mg) | 252 | 17.9* | 582 | 19.1* | 946 | 24.2* | 1030 | 26.6* | **962** | **30.5** | 783 | 34.1 | 571 | 36.6* | 1097 | 46.4* | <.0001 |  |
|  | Magnesium (mg) | 230 | 16.3* | 551 | 18.1* | 873 | 22.4* | 1119 | 28.9* | **1095** | **34.7** | 970 | 42.3* | 684 | 43.9* | 1334 | 56.5* | <.0001 |  |
|  | Iron (mg) ‡‡ | 536 | 38.1* | 1362 | 44.6* | 2060 | 52.8* | 2377 | 61.5 | **2074** | **65.7** | 1612 | 70.2 | 1139 | 73.1* | 1937 | 82* | <.0001 |  |
|  | Zinc (mg) | 392 | 27.8* | 1125 | 36.9* | 1700 | 43.5* | 1710 | 44.2 | **1474** | **46.7** | 1154 | 50.3* | 782 | 50.2 | 1256 | 53.2* | <.0001 |  |
|  | Copper (mg) | 1284 | 91.2* | 2919 | 95.6* | 3801 | 97.4* | 3808 | 98.5 | **3127** | **99.0** | 2272 | 99.0 | 1550 | 99.4 | 2352 | 99.5 | <.0001 |  |
| Nutrients with AI †† | |  |  |  |  |  |  |  |  |  |  |  |  |  |  |  |  |  |  |
|  | n-6 PUFA (g) | 820 | 58.2* | 2047 | 67.1* | 2673 | 68.5 | 2727 | 70.5 | **2223** | **70.4** | 1652 | 72.0 | 1127 | 72.3 | 1693 | 71.6 | <.0001 |  |
|  | n-3 PUFA (g) | 762 | 54.1* | 1769 | 58.0 | 2375 | 60.8 | 2398 | 62.0 | **1944** | **61.6** | 1440 | 62.7 | 1002 | 64.3 | 1478 | 62.5 | <.0001 |  |
|  | Vitamin D (μg) | 405 | 28.8* | 934 | 30.6* | 1330 | 34.1 | 1397 | 36.1 | **1227** | **38.9** | 906 | 39.5 | 669 | 42.9 | 1009 | 42.7 | <.0001 |  |
|  | Vitamin E (mg) | 641 | 45.5* | 1562 | 51.2* | 2185 | 56* | 2400 | 62.1 | **2064** | **65.4** | 1678 | 73.1* | 1166 | 74.8* | 1925 | 81.5* | <.0001 |  |
|  | Vitamin K (μg) | 377 | 26.8* | 1366 | 44.8* | 2329 | 59.7* | 2747 | 71* | **2407** | **76.2** | 1927 | 84* | 1331 | 85.4* | 2152 | 91.1* | <.0001 |  |
|  | Pantothenic acid (mg) | 728 | 51.7* | 1844 | 60.4* | 2586 | 66.2* | 2711 | 70.1* | **2373** | **75.1** | 1844 | 80.3* | 1251 | 80.2* | 2022 | 85.6* | <.0001 |  |
|  | Phosphorus (mg) | 958 | 68* | 2294 | 75.2* | 3166 | 81.1* | 3312 | 85.6 | **2728** | **86.4** | 2073 | 90.3* | 1394 | 89.4 | 2193 | 92.8* | <.0001 |  |

Adherence (%) of estimated energy requirement (EER)-adjusted nutrient intake to the Dietary Reference Intakes for Japanese (2020) by vegetable intake categories, adults >= 20 years, NHNSJ 2016 (n 21606). The column shown in bold is the reference group.

† Based on the tertiles of the number of nutrients (for each tertile, range shown in the brackets) that were not adherent to the DRI. T1: 0-12; T2: 13-16; T3: 17-27.

‡ Nutrients that meet the DG values.

§ Shown as the number (%) of participants with intakes within the recommended ranges.

¶ Shown as n (%) of participants with intakes above the recommended limit.

††Reference values for women were referred to those set for pre-menopausal populations.

*P <0.001 from the reference group (280-<350 g/d) obtained from Wald chi-square test based on multivariate logistic test adjusted for gender (men or women), age (20-29, 30-39, 40-49, 50-59, 60-69, 70-79, and >=80 years), region (Hokkaido, Tohoku, Kanto 1 and 2, Hokuriku, Tokai, Kinki 1 and 2, Chugoku, Shikoku, and Kita- and Minami-Kyushu), weight status (<18.5, 18.5-24.9, >=25.0 kg/m2, and missing), and smoking experience (yes, no, and missing), drinking habits (yes, no, and missing).
